# Technology Effects and Child Health: Wellness Impact and Social Effects (TECHWISE): Protocol for a Prospective, Observational, Real-World Study

**DOI:** 10.2196/69358

**Published:** 2025-06-19

**Authors:** Scott H Kollins, Jessica Flannery, Karen Goetz, Samir Akre-Bhide

**Affiliations:** 1 Aura Sub, LLC Boston, NC United States

**Keywords:** youth, adolescence, social media, mental health, technology use

## Abstract

**Background:**

There has been controversy over the extent to which technology use in general—and social media exposure specifically—may be associated with adverse youth mental health outcomes. To date, studies have generally been small and exploratory, relying on youth self-reports to characterize technology and social media use patterns. The goal of this study is to explore the associations between objectively collected data, gathered through a commercially available app, and youth mental health outcomes.

**Objective:**

The broad goal of this study is to characterize the association between objectively measured technology/social media use and a range of mental health–related outcomes. Three specific aims will be initially addressed. First, we aim to quantify the association between objectively measured smartphone use and measures of well-being. Second, we aim to differentiate types of engagement—specifically, the types of content consumed versus the overall time spent on the device—and examine their association with outcomes. Third, we aim to identify moderating factors, such as age, gender, and socioeconomic status, that might influence these relationships. A secondary broad objective of this research is to establish a freely available data resource that can be accessed by qualified investigators to address a much wider range of questions in the future.

**Methods:**

Up to 1000 male, female, and nonbinary youth aged 8-17 years (inclusive), along with their primary caregivers, will be enrolled in the study. Youth participants must have their own dedicated iOS- or Android-based smartphone or tablet, and both they and their parents must be willing and able to download and install the data collection app on their devices. The study is open to all US-based participants who meet these 2 criteria. Following electronic consent (eConsent), participants and their caregivers will complete a range of baseline measures electronically, including assessments of psychiatric and social functioning, as well as measures of loneliness, digital stress, and disordered eating. Caregivers will be asked to provide information on the participant’s health and mental health history. Youth and caregivers will then complete a similar battery of assessments 1, 2, and 3 months after baseline. Youth participants will also respond to daily questions about their mood, stress, physical activity, and sleep. Both youth and their caregivers will be compensated for completing measures at each time point. The data collection app gathers a wide range of daily data from the participant’s device, including temporal patterns of use, the number and frequency of various app usage, social interactions within apps, and keystroke data. A variety of analytic methods will be used to address key questions related to how technology and social media use are associated with mental health and wellness outcomes.

**Results:**

Enrollment for this study began on November 13, 2024. As of May 20, 2025, a total of 106 participants and their caregivers had consented to participate and provided baseline data. An additional 203 children and parents have consented and are currently undergoing eligibility verification and enrollment. Initial data analysis is anticipated to begin in late autumn 2025 or winter 2026, with the expected publication of initial findings in spring 2026.

**Conclusions:**

This study will be among the largest to date to collect both objective device usage data and validated, clinically relevant outcome measures. In accordance with our data-sharing policies, any qualified investigator will be able to access the study data, provided appropriate steps are followed.

**Trial Registration:**

ClinicalTrials.gov NCT06664944; https://clinicaltrials.gov/ct2/show/NCT06664944

**International Registered Report Identifier (IRRID):**

DERR1-10.2196/69358

## Introduction

Over the past 15 years, there has been a dramatic rise in technology use among children and adolescents, particularly following the introduction of smartphones [1]. Nearly 95% of adolescents report using social media [2]. By age 17, the vast majority of youth in the United States have access to a smartphone and report spending hours on it daily. Nearly 50% say they are on social media and other smartphone-based apps “nearly constantly” [3].

Concurrent with these changing patterns of technology use by young people, there has been widespread concern about increases in youth mental health problems, including self-harm/self-injurious behavior, rates of mood and anxiety problems, and self-reported loneliness. Even in the decade preceding the onset of the COVID-19 pandemic, high school students reported increases in feelings of sadness and hopelessness, along with higher rates of suicidal ideation and behavior [4]. Since 2020, there is considerable evidence that rates of mental health challenges among youth, such as depressive and anxiety symptoms and self-harm, have continued to rise. The scope of these challenges prompted both the US Surgeon General and the American Academies of Pediatrics and Child and Adolescent Psychiatry to issue proclamations calling for a range of measures to address the problems [5,6]. Yet, more than 3 years later, there is little evidence that rates of mental health challenges among youth are abating.

Given the temporal congruence between the rise of technology and social media use and the reported increases in youth mental health challenges, much research and public discussion have centered on how these trends may be related. The link between social media use in particular and adverse mental health outcomes has drawn national attention, including a US Congressional hearing with the chief executive officers of the top social media companies [7], and 2 additional reports from the US Surgeon General specifically highlighting potential harms of social media use on youth mental health [8,9]. Most recently, the Governor of California signed a bill banning social media apps from sending notifications during school hours and late at night, and is considering a bill that would require a mental health warning label on social media apps [10].

Despite the highly publicized concern over the link between tech/social media use and mental health outcomes in young people, the published evidence is mixed. Many studies report associations between social media use and mental health problems [11-13], while others find weak or no relationship—or even a positive link [14-16]. One reason for these mixed findings is almost certainly that the relationship between smartphone use and mental health outcomes is not straightforward. A myriad of person-centered factors (eg, age, sex/gender identity, preexisting mental health condition, reward sensitivity), content exposure (eg, inappropriate material, cyberbullying), and nondevice activities (eg, in-person connection, physical activity) can influence the links between smartphone use and mental health outcomes [17,18].

One common feature of studies examining the mental health impacts of smartphone or social media use on young people is the nature of data collection. The literature in this area typically relies on self-reports of usage patterns by youth themselves or their caregivers [19]. However, the correlation between self-reported social media use and objectively derived use (from the devices themselves) is modest. For example, a meta-analysis of 47 studies found a correlation of 0.35 between self-reported and objectively measured social media use [20]. Given the limitations of data collection in previous studies, there is general agreement—both in the published literature and in reports from the Surgeon General and other public sources—on the need for more objective data to better understand how technology and social media use may influence mental health outcomes in young people.

The overall goal of this longitudinal, observational study is to examine the association between youth technology and social media use and mental health outcomes, including mental health and wellness, physical activity, digital stress, and loneliness, as well as daily ratings of mood, stress, and sleep. Our initial analysis, described in this protocol, will focus on 3 primary aims. First, we aim to quantify the association between objectively measured smartphone use and measures of well-being. Second, we aim to differentiate types of engagement—specifically, the types of content consumed versus the overall time spent on the device—and their associations with outcomes. Third, we aim to identify moderating factors, such as age, gender, and socioeconomic status, that might influence these relationships.

A second broad goal of this research is to establish a data set that can be used by a wide range of investigators to address a variety of questions over time. The specific aims outlined above represent our initial priorities for data analysis, but our study design will support the exploration of a myriad of additional questions.

This research will extend previous work in 3 important ways. First, device usage data will be collected using a commercially available product (the Aura app), which provides an objective measurement of a wide range of youth digital activity, including patterns of time spent on various apps throughout the day and overnight, social interactions, messages sent across platforms, and derived sentiment from outgoing messages. Second, rather than examining the relationship between device usage and mental health outcomes solely at the population level, we will leverage the unique combination of objective smartphone data and daily subjective well-being measures to adopt an idiographic approach. This allows us to use individuals as their own baselines, capturing within-person changes in smartphone use to predict corresponding shifts in well-being over time. Third, the granularity of the data will allow us to identify distinct usage patterns across children and adolescents. By including person-centered factors (eg, age, gender, and race/ethnicity) alongside both device usage and off-device behaviors, the data could help uncover protective and moderating effects within broader behavioral trends. These refined approaches—leveraging both the depth and breadth of objective and subjective data on smartphone use and well-being—aim to clarify this controversial topic and provide a foundation for a wide range of subsequent research, clinical interventions, and policy development.

## Methods

### Ethics Approval

The study is approved by the WCG IRB Copernicus Group Institutional Review Board (study approval number 20243405; approval date: September 25, 2024) and has been registered on ClinicalTrials.gov (identifier NCT06664944). Informed consent and assent will be obtained from all eligible participants. Copies of the institutional review board approval notification, as well as the current versions of the parent consent and child assent forms, are included in Multimedia Appendices 1 and 2, respectively. Although the data for this study are not deidentified, all study-related data (eg, outcome measures, app data) will be stored separately from any identifying information for each participant. Individual-level participant data will not be disclosed in any subsequent manuscript or presentation. Participants will receive US $200 per child and US $100 per parent/caregiver for completing all aspects of the research study, for a total compensation of US $300. In addition, active participants who enroll and complete the baseline assessments are eligible to receive a US $25 bonus for each successful participant referral—up to 10 additional eligible participants who consent, enroll, and complete baseline assessments. This referral program is limited to 10 referrals, for a maximum of US $250 per participating dyad.

### Study Design

**Figure 1:**
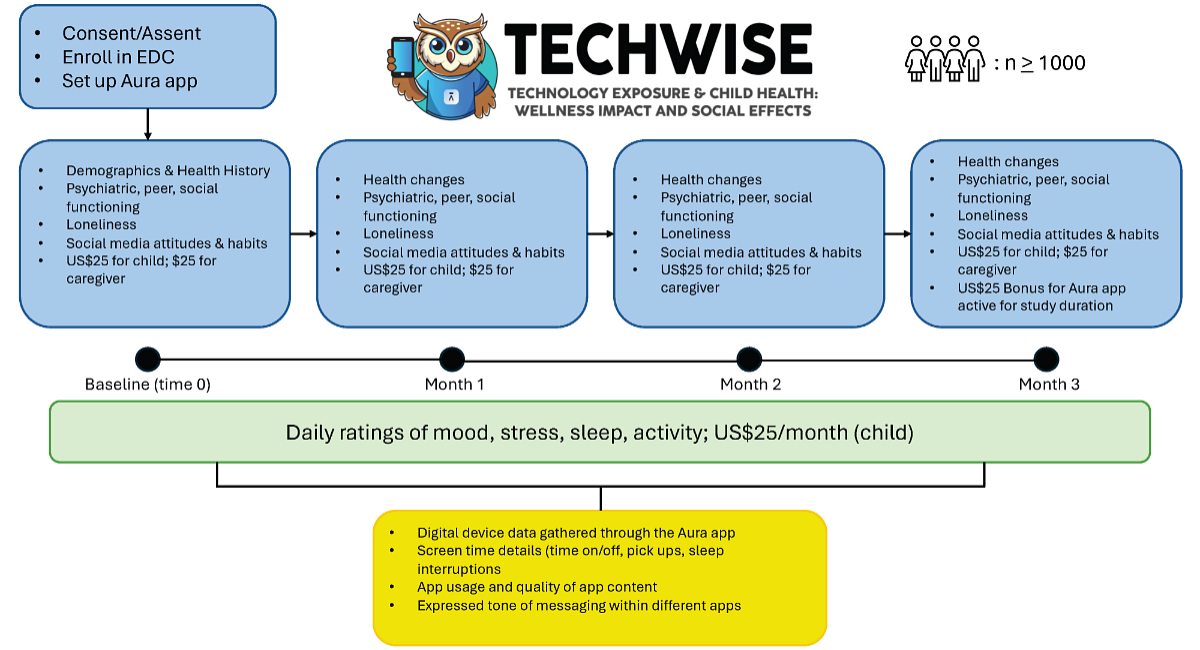
TECHWISE (Technology Effects and Child Health: Wellness Impact and Social Effects) study schematic. EDC: electronic data capture.

This is a prospective, observational study. Figure 1 provides a schematic of the study, including participant incentives.

### Setting

The study will be conducted completely remotely in the United States.

### Participants

The initial enrollment goal for this study is 1000 participants; however, depending on the pace of enrollment, a larger sample may be recruited. Data analysis is expected to begin in the spring or early summer of 2025.

Participants for this prospective cohort study must meet the following eligibility criteria:

- Child participants will be male, female, or nonbinary individuals between the ages of 8 and 17 years (inclusive).
- A parent or authorized legal guardian must be willing to provide informed consent, and the child must be willing to provide assent.
- Both the parent/authorized legal guardian and the child must own a mobile device that either already has the Aura app installed or is capable of installing and running the Aura app and the appropriate parental control features.
  The parent/authorized legal guardian and child must be willing for the child to use a dedicated device for the duration of the study (ie, the device cannot be shared with a sibling or other family member).
  Multiple children from the same family may be eligible to participate, provided that each enrolled child uses a dedicated device, is able to complete outcome assessments independently, and the parent/authorized legal guardian is able to complete assessments for each enrolled child.
- Both the parent/authorized legal guardian and the child must be willing to share data collected through the Aura app and related device apps.
- The consented parent/authorized legal guardian and child must maintain active Aura accounts throughout their study participation (ie, they must activate the appropriate features and keep them enabled for the duration of the study).
- The consented parent/authorized legal guardian and child must be able to follow written and verbal instructions in English, as assessed by the principal investigator or study coordinator.
- The consented parent/authorized legal guardian and child must be able to comply with all testing and study requirements.

Participants will be excluded if they do not meet all of the above inclusion criteria or if they have any medical conditions or social circumstances that, in the opinion of the investigator, may confound study data or assessments. This exclusion criterion is included to address unforeseen circumstances that could impact a participant’s ability to complete assessments or result in atypical device use, potentially leading to misleading data. Any exclusions under this criterion will be reported with the study results.

### Study Recruitment

Given the remote nature of the study, any US-based participant/caregiver dyad who meets the eligibility criteria can enroll via the study landing page [21]. Active recruitment of participants and their parents/caregivers will be conducted virtually across the United States, with efforts focused on enrolling a representative and thus generalizable sample of youth and their parents with respect to race/ethnicity, geographic location, and socioeconomic status. We will monitor enrollment periodically and adjust recruitment efforts as needed to maximize generalizability. Details on the sample demographics and potential limitations related to generalizability will be reported in subsequent papers and presentations. Specific recruitment sources to be used include the following: (1) social media advertisements; (2) current Aura customer base; (3) other selected media activity and announcements (Aura-sponsored marketing activities, Aura Parent Community site (Digital Parenthood [22]), which is free and open to the public; (4) schools/parent-teacher association partnerships; (5) partnerships with academic researchers; and (6) word of mouth.

### Study Enrollment

Interested parents/caregivers and their children will be directed to the study landing page [21]. The landing page will provide detailed information about study participation and enrollment procedures. It will also prominently display the eligibility criteria to help parents/caregivers and children determine their interest and eligibility for the study.

If they affirm eligibility and interest, they will be directed to an electronic consent (eConsent)/assent page. All data collection, including eConsent/assent, will be conducted using a commercially available electronic data capture system (OpenClinica; OpenClinica, LLC). Participants will be enrolled fully remotely (direct-to-participant) and monitored by the central principal investigator (SHK).

Once participants and caregivers assent/consent to participate in the study, they will be prompted to schedule an online visit with the study team (using Google Calendar [Google LLC] and Zoom [Zoom Communications, Inc]) to complete enrollment. This virtual visit will allow study staff and participants to complete the following 4 tasks to verify eligibility and confirm that inclusion criteria have been met: (1) verify parent/caregiver identity; (2) review and countersign consent/assent forms; (3) set up the Aura app on parent and child participant devices; and (4) confirm participants’ preferred methods of contact for study measures. Participants who are unable to schedule a live session will have the option to verify their identity and complete the Aura app setup remotely (ie, by uploading a picture of identification and other materials for identity verification, and sending screenshots of the successfully installed Aura app). Study staff will also be available to answer any questions about study objectives and activities during the remote session or via email or phone.

Participants and their caregivers will receive free Aura app subscriptions for the duration of the study. Current Aura users will be credited with 3 months of subscription time on their accounts.

Parents/caregivers and child participants will also be compensated for completing study-related activities. The total possible compensation over the duration of the study will be US $200 for child participants and US $100 for caregiver participants, amounting to US $300 per caregiver-child dyad. Participants will receive payment for each completed task (see Table 1). Active participants will also be offered an optional US $25 referral bonus, payable to the referring parent, for each new eligible parent-child dyad they refer who subsequently enrolls and completes baseline assessments.

**Table 1:** Summary of participant incentives.

Activity/incentive trigger	Participant	Amount^a^	Timing	Requirement
Baseline forms	Caregiver	US $25	Baseline	Timely completion within the assessment window
Baseline forms	Child	US $25	Baseline	Timely completion within the assessment window
Monthly forms	Caregiver	US $25/month	Months 1, 2, and 3	Timely completion within the assessment window
Monthly forms	Child	US $25/month	Months 1, 2, and 3	Timely completion within the assessment window
Daily ratings	Child	US $25/month	Months 1, 2, and 3	20 or more ratings completed within the month
Maintain active Aura App	Child	US $25	End of the study	App remains active on their device throughout the entire study duration
Maximum total (tasks)	Caregiver	US $100	Study duration	All required tasks complete
Maximum total (tasks)	Child	US $200	Study duration	All required tasks complete
Maximum total per dyad	Both	US $300	Study duration	All required tasks complete

^a^Additional referral bonus: US $25 per referred dyad that enrolls and completes baseline, up to 10 referrals, for a maximum of US $250.

### Participant Withdrawal or Discontinuation

Participants are free to withdraw from the study at any time, for any reason, without prejudice. They will be provided with a study email address and phone number to communicate their decision to discontinue participation.

Participants who are withdrawn or discontinued after enrollment will be sent an exit survey. Those who are ineligible to enroll will receive an email explanation.

### Study Procedures

#### Baseline and Follow-Up Visits (Months 1, 2, and 3)

Following the enrollment visit, baseline assessments will be sent electronically to participants via their preferred method of contact (email or SMS text message; Table 2). Participants and caregivers will receive email or SMS text message prompts to complete online surveys as outlined below. In general, assessments will be completed monthly. Beginning at baseline, child participants will receive daily email or SMS text message prompts to rate their mood, stress, sleep, and physical activity.

Baseline data collection includes (1) sociodemographic information about the child (eg, age, gender, race, ethnicity, education status, individual education plans, school technology and phone policies, and Zip code); (2) sociodemographic information about the caregiver (eg, age, date of birth, for study compensation eligibility, gender, race, ethnicity, highest education completed, and annual income); (3) medical history for the child (eg, current/past mental health diagnoses, current/past other health diagnoses, prescribed medications, over-the-counter medications, current/past other treatments, and behavioral habits [exercise/activity level, nicotine use, alcohol use, caffeine use, and sleep]); and (4) general (“How did you hear about us?” and “If referred, who referred you?”)

Outcome assessments at baseline include Kiddie Computerized Adaptive Testing (K-CAT), The EPOCH Measure of Adolescent Well-Being, The University of California, Los Angeles (UCLA) Revised Loneliness Scale, The Eating Disorder Screen for Primary Care, The Digital Stress Scale (DSS), The Child & Adolescent Social and Adaptive Functioning Scale, The Adolescent Sleep Wake Scale, The World Health Organization (WHO)-5 Well-Being Index, The K6 Psychological Distress Screening questions, The Social Media Screening Questionnaire/HEADS-4 (Home, Education/Employment, Activities, Drugs/Substances, Sexuality, Safety, Suicide/Mental Health, Social Media/Screen Use)—Child version, The Social Media Screening Questionnaire/HEADS-4—Caregiver version, The Social Media Use Scale, and The Child Safety and Well-Being Survey.

The following assessments will be administered monthly after baseline: K-CAT; The EPOCH Measure of Adolescent Well-Being; The UCLA Revised Loneliness Scale; The DSS; The Child & Adolescent Social and Adaptive Functioning Scale; The Adolescent Sleep Wake Scale; The WHO-5 Well-Being Index; The K6 Psychological Distress Screening questions; and changes in health status (new diagnoses, new treatments [medication or otherwise], hospitalizations/emergency department/urgent care visits).

The following measures will be completed at 3 months after baseline: The Eating Disorders Screen for Primary Care, The Social Media Screening Questionnaire—Child version, The Social Media Screening Questionnaire—Caregiver version, The Social Media Use Scale, and The Child Safety and Well-Being Survey.

Measures will be sent electronically via SMS text message or email with unique participant links directing caregivers and participants to the dedicated study site for completion, except for the K-CAT, which will be completed on the Adaptive Testing Technologies platform.

At each monthly time point, parent participants will be asked to verify that the Aura app is active and running on both their device and the child participant’s device. Additionally, the parent will respond to a single 5-point Likert scale question assessing the child’s receptivity to having the Aura app on their device.

**Table 2:** Schedule of assessments.

Schedule	Baseline	Month 1	Month 2	Month 3
eConsent	Parent/caregiver, child	N/A^a^	N/A	N/A
Identity verification and Aura app setup on parent and child participant devices	Parent/caregiver and child	Parent/caregiver and child	Parent/caregiver and child	Parent/caregiver and child
Demographics/health/medical history	Parent/caregiver	N/A	N/A	N/A
Medical/treatment change	N/A	Parent/caregiver	Parent/caregiver	Parent/caregiver
The K-CAT^b^ battery	Parent/caregiver and child	Parent/caregiver and child	Parent/caregiver and child	Parent/caregiver and child
The EPOCH^c^ Measure of Adolescent Well-Being	Child	Child	Child	Child
The UCLA^d^ Loneliness Scale Version 3	Child	Child	Child	Child
The Eating Disorders Screen for Primary Care	Child	N/A	N/A	Child
The Digital Stress Scale	Child	Child	Child	Child
The Child and Adolescent Social Adaptive Functioning Scale	Child	Child	Child	Child
The Adolescent Sleep Wake Scale	Child	Child	Child	Child
The WHO^e^-5 Well-Being Index	Child	Child	Child	Child
The K6 Psychological Distress Screening questions	Child	Child	Child	Child
The Social Media Use Screening Questions	Parent/caregiver and child	N/A	N/A	Parent/caregiver and child
The Social Media Use Scale	Child	N/A	N/A	Child
The Child Safety and Well-Being Survey	Parent/caregiver	N/A	N/A	Parent/caregiver
The daily report (day 0, daily)	Child	Child	Child	Child

^a^N/A: not applicable.

^b^K-CAT: Kiddie Computerized Adaptive Testing.

^c^EPOCH: Engagement, Perseverance, Optimism, Connectedness, Happiness.

^d^UCLA: University of California, Los Angeles.

^e^WHO: World Health Organization.

#### Participant Enrollment and Engagement Support

Participants will receive ongoing communication throughout the enrollment process and at each study time point to provide support. During enrollment and baseline assessments, participants will receive email or SMS text message reminders from the Technology Effects and Child Health: Wellness Impact and Social Effects (TECHWISE) study team regarding parent/child consents, the virtual enrollment visit, baseline surveys, K-CAT paired interviews, and daily ratings for child participants. Throughout the study, the TECHWISE study team will update a community page to provide news, research updates, and study support to enrolled participants. Additionally, the team will distribute a monthly newsletter to maintain participant engagement and keep communication channels open.

Participants may use Google Calendar, Zoom, and the TECHWISE study team email to schedule 1-on-1 support throughout the study. Additionally, parent participants will receive a study tracker at each monthly time point, listing the study activities for both parent and child participants.

#### Digital Activity Data Collection

The Aura product (current version 3.34.2) is a consumer-focused digital security app available to any smartphone user with iOS (Apple Inc) or Android (Google LLC) operating systems. When activated as part of the Family Plan subscription (used in this study), the app continuously monitors and collects data on a child’s smartphone or tablet device usage. The data gathered include, but are not limited to, the following: time spent on the device; specific app usage patterns (including which app, total time, time of day used, etc); contact information (individuals with whom the child interacts through a range of apps and messaging platforms); content consumption (viewing history, search history, etc); and keystroke data (including overall sentiment).

As product updates and releases occur throughout the study, participants and their parents/caregivers will continue participation using the latest version operating on their devices. The commercially available version of the app is used for all data collection in this study, with no modifications. The app’s Privacy Policy and Terms of Service are included in Multimedia Appendix 3.

### Outcome Measures

#### Demographic/Medical History

This form, completed by parents at enrollment, captures a range of information about the child participant, including age, race/ethnicity, sex/gender identity, family composition, household income, school/educational status, and medical/mental health history (including diagnoses and treatment). Additionally, information about family mental health history is collected.

#### Medical/Treatment Change

This form, completed by parents/caregivers at months 1-3, inquires about any changes in medical status or treatment over the past month, including inpatient/outpatient visits and changes in medication or other treatments.

#### The Kiddie Computerized Adaptive Testing (K-CAT)

The K-CAT is a suite of measures validated for assessing depression, anxiety, mania, attention-deficit/hyperactivity disorder, conduct disorder, oppositional defiant disorder, and posttraumatic stress disorder. It is the children’s version of computer adaptive testing (CAT) based on multidimensional item response theory. The K-CAT includes both child self-report and parent ratings. The initial item responses are used to determine a provisional estimate of the child’s standing on the measured trait, which is then used for subsequent item selection. Item Response Theory is a statistical theory and associated methodology that relates a series of item responses (binary, ordinal, or nominal) to 1 or more latent variables (eg, depression). The K-CAT has been validated against semistructured research interviews for youth aged 7-17 years. The full K-CAT takes an average of 7.56 minutes to complete for the child portion and 5.03 minutes for the caregiver portion [23].

#### The EPOCH Measure of Adolescent Well-Being

The EPOCH Measure of Adolescent Well-Being assesses 5 positive psychological characteristics: Engagement, Perseverance, Optimism, Connectedness, and Happiness. It extends Seligman’s PERMA (Positive Emotion, Engagement, Relationships, Meaning, and Accomplishment/Achievement) theory to adolescents, aiming to identify traits that influence well-being in adulthood. The measure consists of 20 items, validated through factor analysis, and has demonstrated good reliability and validity across multiple samples. Respondents rate statements on a 5-point scale, with findings supporting its stability and predictive validity. The EPOCH takes an average of 5 minutes to complete [24].

#### The UCLA Loneliness Scale Version 3

The UCLA Loneliness Scale Version 3 is a psychometrically valid self-report measure of loneliness and social isolation. It is a 20-item inventory that uses a 4-point rating scale (1=never to 4=always) to assess how often a person feels disconnected from others. The scale includes 10 reverse-coded items. The measure is highly reliable, demonstrating strong internal consistency and test-retest reliability over a 1-year period [25]. It has been validated for use with teens and adults and takes approximately 3-5 minutes to complete.

#### The Eating Disorders Screen for Primary Care

The Eating Disorders Screen for Primary Care is a brief, 5-item questionnaire recommended for use in primary care settings and published by the National Eating Disorders Collaboration. Each item is answered with a “yes” or “no” response, and a “yes” response to any item is considered indicative of risk for disordered eating [26].

#### The Digital Stress Scale

The DSS assesses the stress individuals may experience when engaging with social media. Item content is based on a theoretical model of the negative effects of social media use, as well as insights from focus groups. The DSS consists of a 24-item inventory using a 5-point Likert-type scale across 5 factors: Approval Anxiety, Availability Stress, Connection Overload, Fear of Missing Out, and Online Vigilance. The DSS was developed from a preliminary theoretical model of digital stress [27] and validated through exploratory and confirmatory factor analyses [28,29]. It takes an average of 5 minutes to complete.

#### The Child and Adolescent Social and Adaptive Functioning Scale

The Children and Adolescent Social and Adaptive Functioning Scale (CASAFS) was designed to assess the social functioning of young people across 4 domains: school performance, peer relationships, family relationships, and home duties/self-care. The CASAFS is a self-report inventory consisting of 24 items that evaluate social functioning, defined as the degree to which individuals fulfill various roles in their lives. The CASAFS comprises 4 subscales that examine functioning in the 4 key social role areas relevant to children and adolescents. Six items were selected to reflect each of the 4 dimensions of social functioning. Participants are asked to respond to each social functioning item using a 4-point scale: 1 (never), 2 (sometimes), 3 (often), and 4 (always). Family relationship items include a fifth scoring category: “Does not apply to me.” The questionnaire was designed so that the 1-4 ratings on the CASAFS could be summed across the 24 items to provide a total social functioning score ranging from 24 to 96, with higher scores reflecting a greater level of social functioning. Similarly, subscale scores can be calculated, with each ranging from 6 to 24. The internal consistency and 12-month test-retest reliability of the total scale were acceptable. A significant negative correlation was found between the CASAFS and a measure of depressive symptoms, indicating that high levels of social functioning are associated with low levels of depression. The assessment is validated for children aged 10-17 years and is estimated to take 8-10 minutes to complete [30].

#### The Adolescent Sleep Wake Scale

The Adolescent Sleep Wake Scale is a measure of behavioral sleep problems widely used in general adolescent populations and, more recently, in adolescents with specific comorbidities such as depression and chronic pain. The Adolescent Sleep Wake Scale is a 28-item measure scored on a 6-point rating scale that assesses sleep quality across 5 subscales: going to bed, falling asleep, awakening, reinitiating sleep, and returning to wakefulness, with adequate psychometric reliability [31]. The measure is validated for 12-18-year-old adolescents to self-report monthly and takes less than 10 minutes to complete.

#### The WHO-5 Well-Being Index

The WHO-5 is a short questionnaire consisting of 5 noninvasive questions that measure respondents’ well-being. The scale has adequate validity as a screening tool for depression and as an outcome measure in clinical trials. A systematic review of the literature on the WHO-5, conducted in accordance with PRISMA (Preferred Reporting Items for Systematic Reviews and Meta-Analyses) guidelines, yielded 213 articles. The review demonstrated that the WHO-5 has adequate validity and is a sensitive and specific screening tool for depression [32].

#### The K6 Psychological Distress Screening Questions

The K6 is a 6-item screening scale for psychological distress. It was developed, along with the K10, for the redesigned US National Health Interview Survey. The K6 demonstrates good precision in the 90th to 99th percentile range of the population distribution and consistent psychometric properties across major sociodemographic groups. The K6 has a strong ability to discriminate between the Diagnostic and Statistical Manual of Mental Disorders, Fourth Edition cases and noncases, making it suitable for use in general-purpose health surveys [33].

#### The Social Media Screening Questionnaire (Child and Caregiver Versions)

These questions are based on a series developed to systematically assess social media use and misuse in primary care and are recommended by The American Academy of Pediatrics Center of Excellence for Social Media and Youth Mental Health [34]. They extend the HEADS-4 survey, which guides the assessment of Home, Education, Activities, and Drugs in primary care. For the purposes of this study, these screening questions have been adapted to gather information from caregivers. The questions address which apps youth use, the amount of time spent online daily, whether respondents believe the youth spends too much time online, whether social media use impacts self-confidence, and whether the youth has directly experienced cyberbullying or sexual exploitation.

#### The Social Media Use Scale

The Social Media Use Scale is a questionnaire used to measure individuals’ social media use. It includes 22 items divided into 5 dimensions: social interaction, entertainment, information seeking, convenience, and social comparison [35]. The Social Media Use Scale is valid for individuals aged 12-30 years and takes 3-6 minutes to complete.

#### The Child Safety and Well-Being Survey

The Child Safety and Well-Being survey is a 9-question tool used to gather parent or legal guardian perspectives on their child’s technology use, app use, and online safety and well-being. This survey is consistently used across Aura studies on family and child digital media use.

#### Daily Mood, Stress, and Sleep Report

Daily ratings of mood, stress, sleep, and physical activity will be completed by the child. For mood, stress, and sleep, a 0-100 sliding visual analog scale with descriptive anchor points will be used, where lower ratings reflect poorer mood, higher stress, or worse sleep. For the physical activity rating, participants will choose from a 5-point scale reflecting how much activity they engaged in over the past 24 hours: none, 0-30 minutes, 30 minutes to 1 hour, 1-2 hours, and more than 2 hours. At the end of each week, the daily report will include 3 questions asking the child to reflect on mood, stress, sleep, and physical activity over the course of the week.

### Statistical Considerations

#### Specific Aims

Three specific aims will be initially addressed in this work (Textbox 1).

**Specific aims.**

1. Quantify the association
  Determine the strength and direction of associations between objectively measured smartphone use (eg, total screen time, frequency of use, specific app engagement) and self-reported well-being measures (eg, Kiddie Computerized Adaptive Testing [K-CAT], World Health Organization-5 Well-being Index [WHO-5]).
2. Differentiate engagement types
  Evaluate whether distinct aspects of smartphone engagement—specifically, the types of content consumed versus overall time spent on the device—show differential associations with well-being outcomes.
3. Identify moderating factors
  Investigate the extent to which key person-centered characteristics, including age, gender identity, and socioeconomic status, moderate the relationships observed between smartphone usage and well-being.

#### Statistical Analysis: Specific Objectives

##### Elastic Net Regression Modeling

We will employ penalized regression models to predict baseline self-report outcomes for well-being and mental health. Specifically, for each of the following outcome measures—the World Health Organization-5 Well-being Index (WHO-5) total score, the EPOCH Measure of Adolescent Well-Being total score, the K-CAT Depression Scale total score, and the UCLA Loneliness Scale total score—we will train an Elastic Net regression model using device usage features as predictors. To identify statistically significant predictors for each outcome, we will examine the coefficient estimates of the final trained model. Features with nonzero coefficients after Elastic Net regularization will be considered important predictors and explored further in post hoc analyses.

This approach will enable us to identify which specific aspects of smartphone usage are most strongly and statistically significantly associated with self-reported measures of well-being and mental health at baseline, directly addressing specific aim 1. Furthermore, by examining the patterns of significant predictors across the different outcome variables, we can gain insights into whether time- or content-based engagement metrics are differentially associated with well-being, contributing to specific aim 2. Additionally, by including potential moderators, we can begin to address specific aim 3.

The Elastic Net is a linear regression model that combines the penalties of both Lasso (L1) and Ridge (L2) methods. This approach is especially well-suited for data sets with many potentially correlated predictors, as it simultaneously performs variable selection—shrinking coefficients of irrelevant predictors to zero—and manages multicollinearity.

We consider an initial set of 21 features derived from device usage within the first 2 weeks of the study, proximate to the baseline outcome measures. Given the richness of the device usage data, there is no clear upper limit to the number of features that could be developed. However, 21 features were selected based on initial interest, divided between time-on-device metrics and content-specific engagement, in line with specific aim 2. Additional features such as age, gender identity, household income, and racial and ethnic background will be included to address specific aim 3.

Time on device features are (1) total daily screen time, (2) total daytime screen time, (3) length of screen time sessions, (4) number of problematic screen time sessions (2 or more consecutive hours), (5) length of breaks from device usage, (6) number of breaks from device usage, (7) total nighttime screen time, (8) frequency of late-night usage, (9) duration of usage (session lengths), (10) total opportunity for sleep (time offline in the sleep window), and (11) consistency in their nighttime device schedule.

Content-based features include (1) proportion of time spent on 6 categories, namely, social, social generative artificial intelligence (AI), gaming, generative AI, educational, and productivity; (2) frequency of using apps; (3) percentage of time with social AI experience for human-AI interactions; (4) number of messages sent on social platforms; and (5) ratio of consumptive versus interactive social interactions.

For each outcome variable, the Elastic Net model will be trained using 10-fold cross-validation. This robust method partitions the data into 10 equally sized folds. The model is trained on 9 folds and evaluated on the remaining fold, repeating the process 10 times so that each fold serves as the validation set once. This procedure provides a reliable estimate of the model’s generalization performance and helps mitigate overfitting. During cross-validation, we will systematically sweep across a grid of Elastic Net hyperparameters: the mixing parameter (α), which controls the balance between L1 and L2 penalties (ranging from 0 to 1), and the regularization strength (λ), which scales the overall penalty. The optimal hyperparameters will be selected based on the mean squared error performance across the 10 validation folds. After identifying these optimal values through cross-validation, the Elastic Net model will be retrained on the entire data set. The resulting model will provide coefficient estimates for each of the 21 device usage features and other moderating features (age, gender identity, etc) in predicting the respective well-being or mental health outcome. To test for moderation, these additional features and their interaction terms will be included as independent variables in the Elastic Net model during fitting. Variables with nonzero coefficients will be considered significant, and their effects on the dependent variables (scores from the WHO-5, EPOCH, K-CAT, etc) will be further investigated by comparing models with and without these moderating factors.

##### Analysis Populations

The primary analysis population will include all participants enrolled in the registry who complete their baseline assessments and have at least 10 days of device usage data within a 14-day period (ie, up to 4 days of missing data within those 2 weeks). Additional analyses may be conducted on subgroups defined by baseline sociodemographic or clinical characteristics, technology use patterns (eg, high-frequency users, social media users), time of enrollment, and other factors.

##### Sample Size

The initial target enrollment for the study is 1000 participants with complete data for baseline and months 1-3. While we aim to explore a wide range of outcomes related to digital device usage and mental health, our initial analysis will focus on the specific aims outlined above.

To assess the appropriateness of this sample size for powering the planned analyses, we consider our initial set of 21 features. For a multiple regression model (of which Elastic Net is an extension), assuming a moderate effect size (*R*^2^=0.13, Cohen *f*^2^=0.15) between a well-being measure and 21 device usage–based predictors, a sample size of 160 participants is required to achieve 80% power. Our goal in enrolling additional participants in this ongoing study is to enable more fine-grained analyses of other research questions and to provide a resource for qualified investigators to explore a wider range of analyses (see the “Data Sharing” section).

##### Additional Possible Analytic Approaches

Beyond the specific aims noted above, the study design supports a wide range of questions related to the association between device usage and outcomes in youth. This section provides an overview of the general approach to statistical analyses—both quantitative and qualitative—that may be used to analyze the collected data. Given the exploratory, observational nature of the study, the data will allow for a wide range of questions to be addressed. Specific procedures will vary depending on the aims and data used in future publications.

The broad goal of this study is to characterize the association between objectively measured technology and social media use and a range of mental health–related outcomes. The initial specific objectives of the study are as follows:

- To identify objectively measured patterns of technology and social media use in children and adolescents, and to assess whether important individual differences in use patterns over time exist based on various variables. These variables include, but are not limited to, age, sex/gender identity, race/ethnicity, geographic location, and presence of existing mental health diagnoses or treatment.
- To examine bivariate associations between technology use features and mental wellness and mental health outcomes.
- To develop preliminary predictive models using technology use and smartphone-derived parameters to predict point-in-time mental health outcomes, as well as changes over time.

##### Possible Analyses for Objective 1

We aim to identify objectively measured patterns of technology and social media use in children and adolescents, and to assess whether significant individual differences in use patterns over time exist based on various variables.

A range of descriptive analyses will be used to characterize the sociodemographic and other characteristics of the study population. For continuous variables, measures will include mean and SD, as well as median, minimum, maximum, and IQR, as appropriate. Categorical variables will be summarized using counts and percentages. To explore changes in the profile of participants entering the study over time, results may be stratified by enrollment date, and grouped by calendar month, quarter, or year. We will also examine patterns of technology and social media usage as a function of sociodemographic variables (age, sex/gender, etc). Additional analyses (eg, ANOVA, paired *t* tests) may be used to examine differences in usage patterns over time, both within and between participants.

##### Possible Analyses for Objective 2: Examining Bivariate Associations Between Technology Use Features and Mental Wellness and Mental Health Outcomes

Given the wide range of variables derived from the Aura app and the number of assessments collected, all analyses will be considered exploratory. As an initial step toward this objective, we will conduct simple bivariate analyses between technology use variables and mental health outcomes. Example questions include, but are not limited to, the following:

- Is the total device usage time measured at baseline associated with mental health outcomes, such as higher probabilities of mental health conditions, as measured by the K-CAT?
- Do baseline psychological distress (measured by the K6) or well-being (measured by the WHO-5) predict patterns of technology and social media use over time?
- Are temporal patterns of device usage (eg, increased evening or late-night use) associated with sleep or physical activity outcome measures?
- Does digital stress (measured by the DSS) mediate the relationship between device usage and mental health outcomes?

As more participants enroll over time, we will progressively be able to address more complex questions regarding device usage and changes in mental health–related outcomes. Examples of time-related outcomes we may measure include, but are not limited to, the following:

- Are there relationships between variability in daily mood, stress, or sleep and daily patterns of device usage?
- Do broad changes in device usage over weeks or months lead to changes in mental health–related outcomes (positive or negative), or vice versa?
- Are changes in health care utilization (medical events, changes in ongoing treatment, etc) associated with changes in device usage over time?

##### Possible Analyses for Objective 3: To Develop Preliminary Predictive Models Using Technology Use and Smartphone-Derived Parameters to Predict Point-in-Time Mental Health Outcomes, As Well As Changes Over Time

A wide range of analytic techniques will be used to develop and evaluate predictive models aimed at determining whether mental health–related outcomes and their changes over time can be reliably predicted from device usage data. Predictive modeling methods will start with standard statistical approaches (eg, multiple regression) and expand to include nonlinear techniques such as random forests and gradient-boosted regression. With up to daily sampling of some outcomes (mood, stress, and sleep) over a 3-month period and a sample size of 1000 participants, it will also be feasible to train neural network or deep learning models, including temporal models such as long short-term memory neural networks. Example tasks for these prediction models include, but are not limited to, the following:

Do patterns or changes in device-mediated social interactions predict the presence or severity of loneliness?

- Are specific patterns in keyboard data (eg, particular search terms, SMS text messaging sentiment) predictive of changes in mood or anxiety outcomes?
- Are patterns of keyboard activity associated with daily self-reports of mood, stress, or anxiety?
- Can patterns of device usage predict changes in health care utilization over time?

## Results

The Checklist for Reporting Results of Internet E-Surveys (CHERRIES) has been included as Multimedia Appendix 4. Enrollment for this study began on November 13, 2024. As of May 20, 2025, a total of 106 participants and their caregivers have consented to participate and provided baseline data. An additional 203 children and parents have consented and are currently undergoing eligibility verification and enrollment. The anticipated date for initial data analysis is late autumn 2025 or winter 2026, with the expected publication of the initial analyses in spring 2026.

## Discussion

### Anticipated Findings

Despite public interest and academic research on smartphone usage and well-being in youth, critical gaps remain in the literature regarding (1) the scale of real-world data; (2) access to ethically gathered, objective smartphone usage data; and (3) the ability to combine large-scale objective data with validated outcomes to longitudinally assess the association between smartphone use and mental health and well-being.

Together, the strengths of this study design include:

- The ability to recruit a large and diverse sample size through a fully remote and virtual study.
- The ability to ethically collect a wide range of objective smartphone usage data longitudinally from participants who have provided informed consent/assent.
- The ability to combine daily subjective measures of well-being and displacement with objective measures of device usage.

We anticipate that the findings from this study will show that device usage in youth is associated with both positive and negative aspects of mental well-being, depending on the specific usage patterns analyzed (eg, time-based vs content-based) and person-centered factors (eg, age, race, gender). As such, this study design has the potential to clarify some of the inconsistencies previously reported in the literature. For example, while some studies have found associations between excessive social media use and adverse mental health outcomes, such as increased depression and anxiety, others have reported weak or null effects, or even positive associations, such as increased social connectedness [11,36-38].

Therefore, this study presents a unique opportunity to provide real-world insights into the complex relationships between smartphone usage and youth mental health and well-being.

Despite the potential strengths of this research, there are limitations that need to be considered. First, the requirement that youth participants own their own devices may limit the representativeness and generalizability of the sample. This concern is somewhat mitigated by reports indicating that the majority of children aged 11 years and older own a phone, with ownership rates increasing through the teen years and over time [3,39,40]. A second limitation that may affect the generalizability of the findings is that children’s device usage could change as a result of app installation. For instance, parents may choose to implement time limits or content filters, which could restrict the applicability of the results to youth who use their devices without such restrictions. Additionally, given the remote nature of the study, we are unable to verify whether the device on which the app is installed for data collection is, in fact, the child’s primary device. We ask parents and children to attest to this during the consent/assent process; however, we do not independently verify it. As such, this may also affect the generalizability or reliability of the findings. Finally, it should be noted that all authors of this protocol are full-time employees of the study sponsor, Aura, and no independent researchers were involved in the development or implementation of the study. Other tools are available for researchers to gather similar device usage data from youth smartphone or tablet activity—for example, the Effortless Assessment Research System (EARS) [41]—which has been used in previous studies [42,43].

### Dissemination Plans and Future Directions

In addition to the stated goals of the overall study, an objective of publishing this protocol is to provide detailed information about the study design, allowing subsequent peer-reviewed papers and conference presentations to reference it as a source. Beyond the primary analyses described in this manuscript, the collected data will enable a wide range of additional questions and analyses. As described in the “Data Availability” section, the data set will be made freely available to qualified investigators who clearly articulate their research questions and analytical approaches, and who enter into an appropriate Data Use Agreement to ensure participant confidentiality, data security, and integrity.

Given the strong need for data on this important topic, this study is well-positioned to provide valuable new insights into the association between child device usage and mental well-being outcomes.

## Appendix

Multimedia Appendix 1 Institutional review board-approved consent form.

Multimedia Appendix 2 Institutional review board-approved assent form.

Multimedia Appendix 3 Sponsor privacy policy and terms and conditions for app usage.

Multimedia Appendix 4 The Checklist for Reporting Results of Internet E-Surveys (CHERRIES) for internet-based studies.

## Abbreviations

AI: artificial intelligence
CASAFS: Children and Adolescent Social and Adaptive Functioning Scale
DSS: Digital Stress Scale
eConsent: electronic consent
HEADS-4: Home, Education/Employment, Activities, Drugs/Substances, Sexuality, Safety, Suicide/Mental Health, Social Media/Screen Use
K-CAT: Kiddie Computerized Adaptive Testing
PERMA: Positive Emotion, Engagement, Relationships, Meaning, and Accomplishment/Achievement
PRISMA: Preferred Reporting Items for Systematic Reviews and Meta-Analyses
TECHWISE: Technology Effects and Child Health: Wellness Impact and Social Effects
UCLA: University of California, Los Angeles
WHO: World Health Organization

## Data Availability

Data gathered in this study will be made freely available to qualified researchers from academic institutions, provided that appropriate data sharing and usage agreements are executed. Inquiries about data access can be directed to the primary author (SHK; scott@aura.com) or to KG (karen.goetz@aura.com). Alternatively, interested investigators may complete an online form to request access to the TECHWISE data.
