# Supplementary material for: Technology Effects and Child Health: Wellness Impact and Social Effects (TECHWISE): Protocol for a Prospective, Observational, Real-World Study
Source: JMIR Res Protoc. 2025 Jun 19;14:e69358. doi: 10.2196/69358 (PMC12226774; doi:10.2196/69358)
Supplement: Multimedia Appendix 1 [file resprot_v14i1e69358_app1.docx]

# RESEARCH INFORMED CONSENT

**Parent/Legal Guardian Informed Consent Form**

**TITLE:** Technology Exposure and Child Health: Wellness Impact and Social Effects (TECHWISE): An Observational Product Registry Study

**PROTOCOL NO.:** Aura-002

WCG IRB Protocol #20243405

**SPONSOR:** Aura

**INVESTIGATOR:** Scott Kollins, PhD

250 Northern Avenue

Boston, Massachusetts 02110

United States

# STUDY RELATED

**PHONE NUMBER(S):** (866) 579-7576 (Toll Free Study Support)

911 (24 hours)

AuraResearchSupport@aura.com (Study email)

Your participation in this study is voluntary. You may decide not to participate or you may leave the study at any time. Your decision will not result in any penalty or loss of benefits to which you are otherwise entitled.

If you have questions, concerns, or complaints, or think this research has hurt you, talk to the research team at the phone number(s) listed in this document.

**RESEARCH CONSENT SUMMARY**

You are being asked for your consent to take part in a research study. This document provides a concise summary of this research. It describes the key information that we believe most people need to decide whether to take part in this research. Later sections of this document will provide all relevant details.

**How long will I be in this research?**

We expect that your taking part in this research will last up to 3 months.

**Why is this research being done?**

The purpose of this research is to understand if the way children and adolescents use electronic devices affects their mental health or how they feel.

**What happens to me if I agree to take part in this research?**

If you decide to take part in this research study, you and your child/adolescent will need to fill out surveys and use the Aura app to collect information on your child’s device usage, including social media and online games.

**Could being in this research hurt me?**

There is a risk of loss of privacy of your study information.

**Will being in this research benefit me?**

It is not expected that you will personally benefit from this research. Your participation in this research may help to understand if the ways children and adolescents use of electronic devices affects their mental wellness.

**What other choices do I have besides taking part in this research?**

You can use the Aura app without taking part in this research. You do not have to take part in the research.

**What else should I know about this research?**

This study is open only to participants in the United States. You and your child must live in the United States and have United States phone numbers associated with your digital devices (smartphone or tablet).

The study is fully virtual. The study does not require any in-person visits or treatments.

To take part in this research, you and your child must have an active Aura application and membership for the 3-month study duration. If you do not have an existing Aura account, the study will provide one for you at no cost. If you do have an existing Aura account, the study will issue a credit to your account for the 3-month study duration.

Your child must be between 8 and 17 years old at the time of consent and enrollment. Your child must remain under 18 years old during their study participation.

You and your child must have a dedicated device (iOS or Android, phone or tablet) that can run the Aura application. The study will not provide devices.

There is a possibility that your study data might be shared with other researchers without your additional consent. If this happens, there will be no personal identifiers linked to your questionnaire/survey data, so it will not be possible to identify you or your child.

You will not receive the results of the testing done as part of this research.

**DETAILED RESEARCH INFORMATION**

You and your child are being invited to take part in a research study on the association between technology and device usage and a range of mental wellness and other health outcomes. This is a fully remote, observational study. Observational means that the researchers are interested in collecting information on what happens with your and your child during the course of normal daily activities. Your child is not being asked to take any treatments or do anything other than complete forms and use the Aura product.

The purpose of this form is to help you decide if you want your child to take part in the research study. It is important that you read all the information contained in this form and to ask the study staff any questions you may have.

It is also recommended that you watch the informational video content that accompanies this written form.

Please keep a copy of this form for your records. If you have any questions or problems during the study, you may contact the study staff by using the phone number and/or email address on the first page of this form.

# What should I know about this research?

- This is an observational study. It will take place fully remotely, which means you will not have to visit any study sites to participate. You and your child will be asked to complete online study activities.
- Participation in this research is voluntary. Whether you and your child take part is up to you and your child.
- You may take as much time as needed to decide whether your child will take part in this study.
- There will be no penalty to you or your child for choosing not to take part in this study.
- You can agree for your child to take part and later change your mind. There will be no penalty to you or your child for changing your mind about participation at any time.
- If you and your child decide to take part in this research study, you will be asked to sign this consent form. Your child will be asked to check a box on an assent form indicating willingness to participate.
- You will receive a copy of this consent form and your child’s assent form.

# Why is this research being done?

This study is being done to understand how children’s mobile device usage (smartphones or tablets), including social media use and online games, are related to their mental wellness and mental health, as well as some aspects of their physical activity and sleep. This study is available to all children between the ages of 8 and 17 years who reside in the United States. This study requires that enrolled participants - both parents/caregivers and children – use the Aura app that includes the parental controls features. If interested caregivers/children do not have an existing Aura app account and membership, the study will provide a 3-month subscription at time of enrollment at no cost to participants. If interested caregivers/children do have an existing Aura app account and membership, the study will provide a 3-month credit to your existing account.

# How long will my child be in this research?

You and your child will be in the study for up to 3 months following enrollment.

You and your child can choose to stop taking part in the study at any time without penalty. You can choose to remove your child from the study at any time without penalty. If you receive a free version of the Aura app for enrolling in the study and you decide to stop participating, you will still be able to use the product until the current subscription period ends. If you receive an account credit for enrolling and you decide to stop participating, you will still retain that credit on account.

# What happens to me and my child if I agree for me and my child to take part in this research?

If you agree for you and your child to take part in this study, you will be asked to sign this consent form. Even if you agree for your child to take part, your child cannot take part unless they also agree to take part in this study.

At enrollment, you and your child will be asked to participant in an online visit with the study team. This visit will take place on Zoom (an online meeting platform). The purpose of the online enrollment visit will be to help you and your child setup the Aura app, and to answer any questions about the study.

Throughout the study, you will be asked to enable specific features within the Aura app that help to gather information about your child’s device usage. The information collected will include your child’s on and off-line activity including social media, as well as sleep, location and activity levels.

You will be asked to complete study activities at the time of enrollment and then once a month for 3 months. Study activities will include electronic surveys that will ask you and your child about the following:

- Demographic information
- Your child’s medical history and changes in medical issues and treatment over time
- A range of questionnaires about feelings and behaviors that are associated with mental health challenges
- Questionnaires that ask about well being and positive feelings, including relationships with family and friends
- Questionnaires that ask about experiences with social media and device usage
- A questionnaire about feelings of loneliness
- A questionnaire that ask about activity sleep
- Your child will also be asked **daily questions** about their mood, stress, sleep, and physical activity

You and your child will be asked to complete most of the forms at Baseline (right after you and your child agree to participate), and then on a monthly basis over the next 3 months. Several of the questionnaires will only be completed at Baseline, and then 3 months later.

Here is what your child will complete during the study:

- K-CAT® modules (Baseline, Months 1-3)
- EPOCH (Baseline, Months 1-3)
- UCLA Loneliness Scale (Baseline, Months 1-3)
- Digital Stress Scale (Baseline, Months 1-3)
- Child & Adolescent Social & Adaptive Functioning Scale (Baseline, Months 1-3)
- Adolescent Sleep Wake Scale (Baseline, Months 1-3)
- WHO-5 Well-being Index (Baseline, Months 1-3)
- K6 Psychological Distress Screening Questions (Baseline, Months 1-3)
- Eating Disorder Screening - Primary Care (Baseline, 3 months)
- Social Media Screening Questions (Baseline, 3 months)
- Social Media Use Scale (Baseline, 3 months)
- Daily Ratings of Mood, Stress, Sleep, Physical Activity (Baseline, daily throughout the study)

Here is what you (the caregiver) will complete during the study:

- Demographic and Medical History Form (Baseline)
- Medical or Medical Treatment Change Form (Baseline, Months 1-3)
- K-CAT® modules (Baseline, Months 1-3)
- Social Media Screening Questions (Baseline, 3 months)
- Child Safety and Well-Being Survey (Baseline, 3 months)

You and your child will receive prompts to complete the study questionnaires via emails or text messages. Except for the K-CAT modules, all of the study questionnaires will be available on a dedicated study site (hosted by Open Clinica). The K-CAT modules will be sent to you via an email prompt.

Your child will also receive prompts to complete their daily questions.

If you or your child do not respond to the assessment prompts, reminder notifications will automatically be sent.

# Study Plan

If you agree for you and your child to be in this study, you will sign this form before starting any study activities. Your child will have a separate assent form to review with you and to check a box indicating their understanding of the study and willingness to participate.

Contact Aura using the information stated on the first page of this form or through the Aura study website if you have any questions about the study.

# What are my responsibilities if my child takes part in this research?

If your child takes part in this research, you and your child agree to:

- Download and install the Aura app on your and your child’s primary devices; enable parental control features as instructed.
- Agree that your child will be the only user of the device they use that is running the Aura app.
- Remain subscribed to the Aura app for the full time you take part in the study.
- Provide valid email address and/or phone number where researchers may reach you and your child, and to which the study assessments can be sent separately to both you and your child.
- Complete the monthly surveys in a timely manner; and, for your child, complete daily questions on a consistent basis.

# Will it cost me money to take part in this research?

If you do not have an existing Aura account, the study will provide you with an Aura app account and subscription at no cost for the 3-month study duration. If you do have an existing Aura account, the study will provide you with a credit to your Aura account to cover the cost of subscription for the 3-month study duration.

You and your child will also need to use your own mobile devices to participate and these will not be provided as part of the study.

You will be responsible for the costs of Wi-Fi or other internet service in your home or other place of study participation.

# Will being in this research benefit my child?

The information learned from this study may benefit other children and adolescents in the future. We will not share specific results from the questionnaires with you or your child. We will provide ongoing updates on study progress and findings to all participants as the study progresses.

You and your child will receive compensation for your participation in this study, upon completion of study surveys. Details of compensation are outlined below.

# Could being in this research hurt my child?

The risks associated with this study are no more than the risk associated with completing online surveys.

Although study staff will take every precaution to keep you and your child’s information confidential, it is possible that information may be accidentally disclosed to unauthorized persons. Every effort will be made to minimize this risk as much as possible. There may be other risks that are currently unknown.

If your child experiences any changes in health or mental health status during participation, contact your healthcare provider.

**What other choices do I have for my child besides taking part in this research?** You and your child do not have to participate in this study to use the Aura app. Your and your child’s decision of whether to take part in this study will not affect your child’s access to the Aura app and its features. If you received the Aura app for free, you will still be able to use the app until the current subscription period ends if you choose to stop participating. If you received an Aura account credit, you will retain that credit if you choose to stop participation.

# What information is collected and what happens to the information collected for this research?

Here are the types of information that may be collected from this research study, as applicable:

- Demographics information
- Information about medical history and changes in medical and treatment status over the course of the study
- Questionnaire responses
- Information that is gathered through routine use of the Aura app regarding your child’s device usage

Information collected for the purposes of this research study will be kept confidential as required by law. The results of this study may be published for scientific purposes, but we will keep your child’s name and other identifying information confidential, unless required to be revealed by law. Data collected and processed during users’ (including your child’s) standard commercial use of the Aura app is governed by Aura’s Privacy Notice found at: https://www.aura.com/legal/privacy-policy.

Your child’s privacy will be protected in the following manner:

- Information will be collected and stored in a secure database
- Information about individuals will not be made publicly available
- No individual names will be used; unique study ID numbers will be generated
- Only authorized researchers and collaborators will have access to individual identifiable data
- Your child’s identity will remain confidential even if the study results are published

Your child’s survey responses and daily ratings will not be shared directly with you.

The information collected in the study may be used in future studies without additional permission from you. This may include research done by other researchers. The information that may be shared will not contain any information that could identify your child. There may still be a chance that someone could identify your child, but this is not likely. The study results will also be made public. These results will not have any information that could identify your child.

Your and your child’s information relating to this study may be shared with individuals and organizations that conduct or watch over this research if they choose to access or inspect records. These entities include:

- the Sponsor, including persons or companies working for or with the Sponsor
- the Institutional Review Board (IRB) that reviewed this research. The IRB is a group of scientists and non-scientists who review the ethics of research. The goal of the IRB is to protect the rights and welfare of study subjects
- Department of Health and Human Services (DHHS) agencies
- Other regulatory agencies

All individuals and organizations listed above are expected to provide you and your child with the same confidentiality as provided by the study staff.

The Sponsor, including persons or companies working for or with the Sponsor, may contact you about other research opportunities. Participation in research is always voluntary, and you do not have to agree to participate in this or any other study. If at any time you decide you no longer wish to be contacted about other research opportunities, you can contact the study team and request to be removed from the list. Aura will never share your contact information with other researchers or studies without your expressed permission.

# Who can answer my questions about this research?

If you have questions, concerns, or complaints about the study, contact Aura at the phone number(s) listed on the first page (866-579-7576), or email the support line listed on the first page of this form (AuraResearchSupport@aura.com).

This research is being overseen by an Institutional Review Board (“IRB”). An IRB is a group of people who perform independent review of research studies. You may talk to them at 855-818-2289 or email them at clientcare@wcgclinical.com if:

- You have questions, concerns, or complaints that are not being answered by the research team.
- You are not getting answers from the research team.
- You cannot reach the research team.
- You want to talk to someone else about the research.
- You have questions about your child’s rights as a research subject.

A description of this clinical trial will be available on http://www.ClinicalTrials.gov, as required by U.S. Law. This Web site will not include information that can identify you. At most, the Web site will include a summary of the results. You can search this Web site at any time.

# Can my child be removed from this research without my approval?

The person in charge of this study can remove your child from this study without your approval. Possible reasons for removal include:

- Study terminated by Sponsor
- Study terminated by another regulatory agency
- If any clinical adverse event (AE), or other medical condition or situation occurs such that continued participation in the study would not be in the best interest of your child
- Child turns 18 while enrolled in the study, and before the 12 month study duration has completed
- Parent and/or child stop using the Aura app with parent controls and keyboard features

We will tell you about any new information that may affect your child’s health, welfare, or choice to stay in this research.

# What happens if I agree for me and my child to be in this research, but I change my mind later?

If you or your child decide to leave this research, contact the research team. Your or your child’s decision to stop participating in this study will not affect your child’s access to the Aura app for the duration of the subscription period.

# Will I be paid for taking part in this research?

For taking part in this research, you will receive compensation for the completion of surveys and questionnaires at each time point. You and your child will each receive $25 for completing all of the Baseline and monthly assessments. Your child will also receive $25 for each month in which they complete at least 20 of their daily reports. If your child completes all measures for the 3 month duration of the study, they will be eligible to earn up to $200 for participation. If you complete all measures for the 3 month duration of the study, you will be eligible to earn up to $100 for participation. The total possible payment for parent and child participation for the 3 month study is $300.

Payment will be sent to you upon the completion of surveys at each timepoint (baseline and months 1-3). Payment will be sent to you in the form of an electronic credit/debit card. Please allow up to 10 business days for payment processing and delivery.

Prior to the first payment, you may choose to provide contact information and to authorize payment to yourself and/or your child. If you decide not to authorize payment, you may decline compensation. Your decision to authorize payment or decline compensation does not affect you and your child’s ability to participate in the study.

You may earn additional money for referring participants to the study. For each referral you make that results in a new parent-child enrollment, you will be paid $25. Referrals will be paid upon successful completion of the parent and child baseline surveys. You are eligible to be paid for up to 10 successful referrals (up to $250).

# Financial Disclosure

Dr. Kollins owns a private interest in the sponsor company. Please feel free to ask any further questions you might have about this matter.

# Statement of Consent:

- I have read and understood the details of the study above, and I acknowledge the details and conditions set forth above
- I understand that participation in this research is voluntary
- I understand whom to contact if I have questions, to talk about problems, concerns, or suggestions related to the research, or to obtain information or offer input about the research
- I understand that my child and I can withdraw at any time without penalty
- I authorize the collection, use, and disclosure of my and my child’s information as described above to the people or groups identified in this consent form for the purposes described in this document
- I will have access to a signed and dated copy of this consent form and the child participant assent form
- I voluntarily agree to participate in and allow my child to participate in this research study
- I state that I am the true parent or legal guardian of the participant
- I state that my child is between the ages of 8 and 17 at the time of consent and study enrollment
- I understand that study participation requires an active Aura app account on both mine and my child’s device, with parent controls enabled and VPN and keyboard detection on
- I understand that study participation requires both me and my child to complete study questionnaires on a timely basis
- I understand that by signing this document, I agree with the statements set forth above and am providing permission for myself and my child to participate in this research
- Child assent is required unless the investigator determines that the capability of the child is so limited that the child cannot reasonably be consulted. Please review the PEDIATRIC PARTICIPANT Assent Form with the child who is capable of assent prior to consenting below.
- By selecting this checkbox, I attest that I am a Legally Authorized Representative (Parent or Legal Guardian) signing on behalf of the Child Participant. My signature documents permission for me and the child/adolescent named in the assent form to take part in this research.
- All children are required to assent.
- If assent is obtained, have the Parent/Legal Guardian or child document the child’s assent on the assent form.

**Date**

**Full Name of Parent/Guardian**

**Full Name of Child/Adolescent**

**Signature of Parent/Guardian**

**Documentation of Child/Adolescent Agreement to Take Part:**
I have explained the study to the child/adolescent and the child/adolescent has agreed to take part in the research and has signed the assent form (if able)

**Signature of Parent/Guardian**

**Date**
