# Supplementary material for: Technology Effects and Child Health: Wellness Impact and Social Effects (TECHWISE): Protocol for a Prospective, Observational, Real-World Study
Source: JMIR Res Protoc. 2025 Jun 19;14:e69358. doi: 10.2196/69358 (PMC12226774; doi:10.2196/69358)
Supplement: Multimedia Appendix 2 [file resprot_v14i1e69358_app2.docx]

**PEDIATRIC PARTICIPANT Assent Form**

**TITLE:** Technology Exposure and Child Health: Wellness Impact and Social Effects (TECHWISE): An Observational Product Registry Study

**PROTOCOL NO.:** Aura-002

WCG IRB Protocol #20243405

**SPONSOR:** Aura

**INVESTIGATOR:** Scott Kollins, PhD

250 Northern Avenue

Boston, Massachusetts 02110

United States

**STUDY RELATED**

**PHONE NUMBER(S):** (866) 579-7576 (Toll Free Study Support)

911 (24 hours)

AuraResearchSupport@aura.com (Study email)

You are being asked to take part in a research study on your feelings and behaviors and how they relate to your digital device (Smartphone and/or tablet) usage. This study asks you to tell researchers about your thoughts, feelings and behaviors, and your patterns of device usage. The study will also ask your parent/caregiver about their observations of your behaviors and device usage. Before you decide if you want to be in this study, we want to tell you about it. This is so you can ask questions and make your decision.

If you want to read more about the study, you can ask for a copy of the form that your parent or legal guardian will read and sign.

You can also watch the video on the study website that describes what it is about and what you are being asked to do as part of it.

# WHY IS THE STUDY BEING DONE?

This study is being done to understand how children’s mobile device usage (smartphones and tablets), including social media use and online games, is related to their mental well-being, as well as their physical activity and sleep. This study is available to all children whose parents/caregivers are using the Aura app that includes the parental controls features.

**AM I ELIGIBLE TO PARTICIPATE?**

You and your parent are eligible to participate in this study if you meet all of the following requirements:

- You are between the ages of 8 and 17 years old at the time of enrollment
- You have a dedicated device (smartphone or tablet) that you will use for the study
- You and your parent reside in the United States with US-based phone numbers
- You are willing to install and run the Aura app on your device throughout the study

# WHAT WILL I BE ASKED TO DO?

The study is 3 months long.

If you and your parent/caregiver agree to participate in this study, you will be asked to agree to what is written in this form. If you are able, you may also be asked to sign this form. Even if your parent or guardian agrees for you to be in this study, you can only be in the study if you also say you want to be in the study. You can decide not to be in the study and you can decide to leave the study before it is over if you want to.

Throughout the study, your parent/caregiver will install the Aura app on your primary digital device (Smartphone or tablet). This app allows your parent/caregiver to receive information about your device usage, including the apps you are using, and the times you use them. The sponsor will have access to this information during the study and will also get information about where you are, your sleep, and how much you move. Your parent will also answer surveys about you.

You will also be asked to complete surveys throughout the study. You will receive an email or text prompt each day that will ask you to complete questions about your mood, stress level, sleep, and physical activity. Once a month, including right after you agree to participate in the study, you and your parent/caregiver will also be asked to complete some longer questionnaires that will ask you about the following:

- Information about you like age, gender, school, and some things about your family
- Information about your health, including visits to the doctor
- Feelings and behaviors that you might experience
- Information about your feelings and experiences using social media and other parts of your online behavior

You will complete all of the study surveys on a web-based site. You will get an email or text notification when it is time for you to fill out your questionnaires, including your daily questions about mood, stress, sleep, and physical activity. If you aren’t able to fill out your questionnaires right away, you will get reminders.

# CAN BAD THINGS HAPPEN TO ME DURING THE STUDY?

This study involves answering a few short questions each day, and then longer questionnaires once a month. Some of the questions might be personal or sensitive, but most of them will be similar to the kinds of things you might be asked about by your parents/caregivers, teachers, or doctors.

Tell your parent or legal guardian or the study staff at the phone number or e-mail on the first page of this document if you want to stop being in this study, or if answering any of the questions makes you feel uncomfortable. You do not have to answer questions if they make you uncomfortable.

# WILL BEING IN THE STUDY HELP ME?

We do not think that being in this study will help you. You and your parents will not get results or information from the surveys. We hope that the information we learn from his study will help other children and their parents/caregivers by learning more about the relationship between device usage and thoughts, behaviors, and feelings.

**WILL I BE PAID?**

If you complete your daily questions and monthly questionnaires on time, you can earn money. The amount of money you can receive is based on the number of surveys you finish. More information is in the consent form your parent is signing. You can have a copy of that consent form to see how much you will be paid.

Additionally, you and your parent may refer friends and family to the study. You will be paid $25 for each referral that results in an enrolled parent-child participant pair (dyad) and completes their baseline. The limit per family is 10 referrals for $250.

# WHO WILL BE ABLE TO SEE MY ANSWERS TO THE QUESTIONS I ANSWER?

Only the research team will be able to see all of the answers to the questions you complete. They will not be shared directly with your parents/caregivers.

# DO I HAVE TO BE IN THE STUDY?

You do not have to be in this study. You can stop at any time. No one will be upset with you or your parents/caregivers if you decide you do not want to be in the study. If you decide to participate, but then change your mind later, this is OK and you should tell your parent/caregiver or you can call or e-mail the study staff at the number or address on the first page of this consent.

**Subject Signature**

# PEDIATRIC PARTICIPANT ASSENT:

I understand that to take part in this study, I agree to the following:

- I have read this form or had it read to me.
- I don’t have to be in this study if I don’t want to.
- I can stop at any time, and no one will be upset with me.
- I have asked any questions I have so far about the study.
- My questions have been answered.

**Date**

**Full Name**

**Signature**
