# Supplementary material for: Technology Effects and Child Health: Wellness Impact and Social Effects (TECHWISE): Protocol for a Prospective, Observational, Real-World Study
Source: JMIR Res Protoc. 2025 Jun 19;14:e69358. doi: 10.2196/69358 (PMC12226774; doi:10.2196/69358)
Supplement: Multimedia Appendix 3 [file resprot_v14i1e69358_app3.pdf]

Supplemental Material

The following are the Privacy Policy and Terms and Conditions that are part of the Aura app that is used to gather device usage data in this study. More general information about the app can be found at aura.com/online-balance, including a more succinct description of our approach to maintaining child privacy ("How does Aura protect my child's privacy?").

Privacy Policy (available at: https://www.aura.com/legal/privacy-policy)

Privacy Policy

Last Updated:
October 3, 2024

English
—
Français

1. What Information Do We Collect About You?2. How Do We Use Your Personal Data?3. Who Do We Share Your Information With and Why?4. Tracking Technologies & Cookies5. Security6. Cross-Border Data Transfers7. Data Retention8. Your Rights with Your Personal Data9. Legal Basis for Our Processing of Your Personal Data10. Your State Privacy Rights (US Only)11. Technology Licensing12. Age Restrictions13. Privacy Policy Updates14. Contact UsArchived Versions

Aura Sub, LLC and its affiliates (collectively referred to as "Aura", "We", or "Us") is a leading provider of digital security products for consumers. This General Privacy Policy describes how we collect and process personal data provided to Aura in connection with your use of our products, services, apps, and websites (including when you are a prospective customer, customer, or former customer ("Customer")) that link to this policy (collectively as our "Services"). The term "personal data" refers to information that is related to an identified or identifiable natural person (aka 'personal information' or 'personally identifiable information').

Product Privacy Notices. This policy describes Aura's general approach to ensuring privacy considerations across all our services. Some of the features within the Aura' Services, however, require Aura to collect and process different personal data from what is described in this policy. The following links provide further information on product-specific features:

1. Aura Identity Theft Protection Services
2. Aura AntiVirus
3. Aura VPN
4. Aura Call Protection
5. Aura Parental Controls

1. What Information Do We Collect About You?

This section describes the various categories and types of personal data we collect from and about you when you use our website, apps, and when you interact with us in relation to our Services. The personal data we may collect may differ depending on which product you use. Please see our Products Privacy Notices for more information. The information we collect includes:

1.1 Personal data you provide to us

We and our service providers may collect personal data about you to provide our services, respond to your requests, and manage your account with us. We collect this information via forms on our websites and apps, when you contact us or register an account, and in our communications with you. Where service components go beyond the base service, we provide you with clear choices to decide if you want these additional functions.

1. Account information. We collect data when you create or update your account. This may include your name, username, email address, phone number, password, your government ID numbers (when you use our Identity Protection Products) and certain other information from you, including if applicable, your account and login information with our affiliate Circle. For our identity protection products, we will also request your Social Security Number (excluding Social Insurance Numbers in Canada) and other personal data.

2. Billing and payment information. To purchase a service, we may collect certain details such as billing name, billing contact details (street addresses, email addresses), and payment instrument details.

3. Identity verification information. To verify your identity, we may collect personal data such as your full legal name, email addresses, your date of birth, social security number, home address, or phone numbers.

4. Communications and submissions. We collect data when you communicate with us (e.g. via email, phone, or chat for support or to inquire about our services), including when you fill out an online form, respond to surveys, provide feedback, post comments to our website, participate in promotions, participate in forums, websites and related information services to share your experiences or discuss technical issues, or submit information through our services.

5. Photos and Documents. You may choose to upload and provide us with images and files that are stored on your device in relation to your use of our Services.

6. Marketing and Advertising Data. We collect data on your participation in promotions, survey responses, your marketing choices, information regarding your preferences based on your use of our services, your interactions with advertising and marketing

communications, and information regarding your customer profile. This may include demographic information such as age and gender.

7. Emails. If you activate our email protection services and connect your email service provider inbox to Aura, we will collect the emails in your inbox and emails that you receive on an ongoing basis. We will collect all data associated with the emails except for any files attached to your emails. This includes, but is not limited to, the sender’s email address, the content of the email, and the subject line.

8. Product-Specific Information. Some of our products, such as our Parental Controls software, may require you to provide us with third party account-specific information such as credentials to access child-specific social media accounts.

1.2 Information collected automatically when you use our services

We automatically collect personal data about your use of our services to monitor for problems and look for opportunities to make improvements. Some of this personal data is collected using cookies and similar technologies (see below for more information).

1. Usage information. We collect information about how you interact with our services, such as how often you use our services, how much bandwidth you use, and when and for how long you use our services.

2. Device information. We collect information such as device identifiers (like IP address or mobile device identifiers), browser types, device types and settings, device manufacturer and model, operating system versions, mobile, wireless, and other network information (such as internet service provider name, carrier name and signal strength), and application version numbers.

3. Diagnostic information. We may collect information about the nature of the requests that you make to our servers (such as what is being requested, information about the device and app used to make the request, timestamps, and referring URLs).

4. Location information. Unless otherwise expressly stated in product-specific materials, we do not automatically collect your precise geo-location information based on your device’s GPS or other device sensor data. However, we may collect your approximate location by calculating an imprecise latitude and longitude based on your IP address to provide you with better service (e.g. to connect you to the nearest and fastest VPN server).

1.3 Information provided to us by third parties

As part of our services, with your consent or where authorized by law, we can receive personal data about you from third parties. We take the same level of precautions and transparency of use that we provide for personal data you provide us directly.

1. Referrals. If you are invited to use an Aura service, the person who invited you may submit information about you, such as your email address or other contact information.

2. Third Party Accounts. Some services may allow you to register an account using a third-party account (such as a Google or Microsoft account), and in some cases, you may register third party accounts such as your bank accounts or social media accounts with us. If you do so, that third party may send us some information about you that they have. In the case of our Parental Controls software, we may receive information associated with account-specific social media content or messaging. You may be able to control what information they send us via your privacy settings for that third party account.

3. Threat Information. We receive information from reputable members of the security industry who provide information to help us to provide, develop, test, and improve our services (for example, lists of malicious URLs, spam blacklists, phone number blacklists, and sample malware). Some of this information may contain personal data on an incidental basis.

4. Business Customers. Organizations that use our business and enterprise products may submit personal data to facilitate account management and invite individuals to use those products. We process such personal data at the direction of such business customers.

5. Monitoring Services. For some of our products and services that we provide to you, we will collect publicly available personal data from third parties to provide the extent of disclosures to you. For example, court records, home title, auto title, and dark web scanning.

2. How Do We Use Your Personal Data?

Aura uses your information for the purposes described below. Aura employs internal risk management functions to ensure we continue to only use your personal data for the purposes we disclose to you and are taking appropriate steps to protect that personal data from exposure. Notwithstanding any other legal bases for the processing of personal data outlined in this Privacy Policy, namely Aura’s legitimate interest, we will only process personal data of residents of Canada with their consent, or where authorized by law.

1. To provide, maintain, troubleshoot, and support our services. We use your personal data for this purpose on the basis that it is required to fulfill our contractual obligations to you. Examples: using information about how much bandwidth you use and how long you use our services in order to provide the services in accordance with a plan to which you have subscribed; using threat and device information to determine whether certain items pose a potential security threat; and using usage information to troubleshoot a problem you report with our services and to ensure the proper functioning of our services.

2. For billing and payment purposes. We use your information in order to perform billing administration activities and process payments, which are required to fulfill our contractual obligations.

3. To communicate with users and prospective users. We use your personal data to communicate with you via email, SMS, push notifications or other messaging about the services or relevant updates, including by responding to your requests, and sending you information and updates about our services. We may do this in order to fulfill our contract with you, because you consented to the communication, or because we have a legitimate interest in providing you with information about our services.

4. To promote, operate or improve our services and advance our or a third party's legitimate interests. We want to offer you the best services and user experiences we can. Where applicable, we have a legitimate interest in continually improving and optimizing our services. To do so, we use your personal data to help ensure the effective delivery of our services and communications to you as well as to our other customers and partners. Examples of when we may use your personal data for this purpose:

- to notify you about changes to our terms or this Privacy Policy;
- to promote and administer co-branded offers with trusted partners;
- enable participation in interactive features of our services;
- communicate commercial promotions or inform you about additional services or features that we think you may be interested in;
- confirm sales conversions and conduct lead generation activities;
- analyze certain usage, device, and diagnostic information to understand aggregated usage trends and user engagement with our services (and, for example, invest in technical infrastructure to better serve regions with increasing user demand);
- use device and threat information or data to conduct spam, scam, threat, fraud and other scientific research to improve our detection capabilities;
- improve our machine-learning algorithms to enhance our services;
- review customer feedback to understand what we could be doing better;
- use information such as who or what referred you to our services to understand how effective our advertising is;
- use information to administer promotional activities such as sweepstakes, contests, offers, promotions and referral programs.

5. For measurement, research and analytics, including to develop new services. Where applicable, we have a legitimate interest in using your personal data for measurement, research and analytics, including to plan for and develop new services. For example, we may analyze certain usage information to understand how users interact with our services and make improvements; we may use customer feedback to understand what new services users may want.

6. For advertising and marketing our services, including targeted advertising, through the use of cookies. For more information on targeted advertising and cookies, please see Section 4 (Tracking Technologies & Cookies) and our Cookie Policy. Our app is not

ad supported, and we do not share personal data from our app with third party advertising services.

7. Aggregated or anonymized data, for any purpose where the information is aggregated or anonymized so that no individual data is directly, or indirectly, identifiable.

8. To prevent harm or liability. We may use information for security purposes (such as to investigate security issues or to monitor and prevent fraud) and to prevent abuse. We may do this to comply with our legal obligations, to protect an individual's vital interests, or because we have a legitimate interest in preventing harm or liability to Aura and our users. For example, we may use account, usage, and device information to determine if an entity is engaging in abusive or unauthorized activity in connection with our services.

9. For legal compliance. We internally use your information as required by applicable law, legal process, or regulation. To learn about our practices regarding sharing your information with third parties for legal compliance purposes, see Section 3.1 below. We also use your information to enforce our legal rights and resolve disputes and complaints.

REMOTE SUPPORT

If applicable for the Parental Control Services, Aura or its duly authorized support representatives, which may include its affiliate Circle (defined below), may need to access your account information in order to troubleshoot, debug, and otherwise offer support and solutions to users. Remote access may be enabled by the user via the App. If you make a support request and wish to limit the level of access for the Parental Control Services in your App or account, you must state those limitations at the time of your support request.

3. Who Do We Share Your Information With and Why?

In some situations, Aura may share your information with third parties who may collect, store, use, process and transfer the data for Aura. Aura employs oversight processes and controls to the secure sharing of personal data with only trusted parties.Neither Aura, nor any of the companies that comprise Aura, sell your personal data. Some of our uses of cookies and/or pixels, however, may be considered a “sale” under California law.

3.1. In General

We may disclose your information in the following circumstances:

1. In accordance with your instructions or consent. For example, some services may allow you to register an account using a third-party account (such as a Google or Microsoft account). If you choose to do so, we will share information with the third-party account provider.

2. To your business organization (for our business services). If a business customer is providing you with access to our services through a business account, others in that organization may be able to see and manage your basic account details and the information associated with your account (such as an administrator). For clarity, we do not share the information inside your account such as bank account information, credit scores, etc.

3. For collaborating with others. Some services may provide ways for different Aura users to interact or collaborate with each other (e.g. family plans). Your information will be shared in connection with those activities if you choose to engage in them.

4. Affiliates. We may share your name, email address, and other contact information with our subsidiaries and affiliates including Circle Media Labs, Inc. ("Circle") to better market our collective products and services to you. We and our affiliated entities may also share information with third-party data controllers where such sharing is legally required, such as with the use of certain cookies and related tracking technologies for compliance with specific geographic laws.

5. With our partners. We may provide your personal data to partners to confirm your eligibility for joint or co-branded offers or to communicate and administer such offers (e.g. verify eligibility, assess effectiveness of joint offer, etc.). Our partners are not allowed to use any data including personal data that they receive from us for any purpose except for communicating, evaluating, improving, and administering the offer in question. This will not affect the partner's ability to use personal data that it may already have obtained from you or other sources. If you do not wish to receive promotional emails from our partners, you can unsubscribe directly using the unsubscribe link or tool provided in the partner's email or other communication to you.

6. Third-party service providers. With the exception of any data collected through your use of our virtual private network where our VPN Product Privacy Notice applies, we may disclose the information we collect to service providers to help us provide some aspects of our services. We work with trusted third parties and partners (including our affiliate companies such as Circle when they act as our service providers). In cases where we may share your personal data, we enter into appropriate confidentiality and data processing agreements with these third parties, review their security practices, and limit information sharing to the scope of what they are helping us with. Examples of activities that third parties help us with include:

- processing customer payments
- providing analytics about our services to help us understand how the services are being used
- providing sales and customer support
- maintaining the infrastructure required to provide our services
- delivering our marketing and advertising content
- Improving our artificial intelligence software

7. For security research purposes. A de-identified subset of our threat intelligence data may be shared with selected reputable members of the cybersecurity industry for the purpose of security threat research and facilitating community efforts to improve online security.

8. In connection with a business transaction. If a corporate transaction occurs, for example changes to ownership or control of all or part of our services, assets, or business, sale of a website, initial public offering, or an investment entity is conducting due diligence in connection with an acquisition or investment, then we may share personal data necessary to that corporate transaction.

9. Aggregated or de-identified data. We may aggregate and de-identify any data we collect and use and share such data so that it no longer reveals the identity of an individual user for regulatory compliance, research and analysis, our own marketing and advertising activities and other legitimate business purposes.

10. To comply with legal process and the law. If you use our VPN product, we help protect your privacy by ensuring that we do not log or record online activities that you conduct over a VPN connection in any way that can be tied back to you, meaning that we do not have any data to share with law enforcement and government agencies who make requests for information about what you were doing through a VPN connection. Subject to the foregoing, we may share your information if we are required to do so by applicable law; to comply with our legal obligations; to comply with legal process; and to respond to valid law enforcement requests relating to a criminal investigation, or alleged or suspected illegal activity that may expose Aura, you, or any of our other users to legal liability. If we share your information for these purposes, we limit the information shared to what is legally necessary, and challenge information requests that we believe are unlawful, overbroad, or otherwise invalid.

11. To enforce our rights and prevent fraud and abuse. We may share limited amounts of your information to enforce and administer our agreements with customers and users, and to respond to claims asserted against Aura. We may also share your information in order to protect against fraud and abuse against Aura, our affiliates, users and others.

4. Tracking Technologies & Cookies

For more information on Tracking Technologies and Cookies and how we use Tracking Technologies and Cookies please see our Cookie Policy.

5. Security

Securing personal data is an important aspect of protecting privacy. Aura employs a range of administrative, organizational, technical, and physical safeguards designed to help protect your data against unauthorized access, use, disclosure, loss, or modification. We endeavor to use reasonably available state-of-the-art network and information security standards, protocols and technologies, including encryption,

intrusion detection and data loss prevention, and we monitor our systems to ensure that they comply with our security policies.

We implement physical, technical and organizational safeguards to protect your personal data under our control, both at rest and in transit, and should these measures fail to prevent a data breach, we will promptly take the necessary remedial measures, and we will provide notices as required by applicable law.

If you have any questions about the security of your personal data or the security of our products, or wish to report a potential security issue, please contact security@aura.com. When reporting a potential security issue, please describe the matter in as much detail as possible and include any information that might be helpful.

6. Cross-Border Data Transfers

Aura may transfer your personal data to regions other than the one in which you reside. We do this to facilitate our operations, and transferees include other Aura group companies, service providers, and partners. Laws in other countries may be different to those that apply where you reside. For example, personal data collected within Canada, Switzerland, the United Kingdom, or the European Economic Area (EEA) may be transferred and processed outside Canada, Switzerland, the United Kingdom, or the EEA for purposes described in this policy.

By submitting information to us, you consent to the communication of your personal data to, and the storage of that data, outside of your region. When such data is outside of your region of residence, it is subject to the laws of that jurisdiction, and may be subject to disclosure to governments, courts, law enforcement or regulatory agencies pursuant to local laws.

When required by applicable law, we put in place appropriate safeguards that help to ensure that such data receives an adequate level of protection. These safeguards may include implementing the European Commission’s Standard Contractual Clauses for transfers of personal data between us and our business affiliates and associates to which we choose to transfer the information that requires these companies to safeguard personal data they process from the EEA, the UK and Switzerland. You may contact us if you would like more information about such safeguards. We implement similar appropriate safeguards with our third-party service providers and further details can be provided upon request.

You are welcome to contact us to obtain further information about Aura’s policies regarding service providers outside of Canada. See Contact Us below.

If you change your region of residence, the Aura affiliate or company responsible for your data may change accordingly, and your data may be transferred to that other company.

7. Data Retention

Aura generally retains your personal data for as long as is needed to provide the services to you, as specified with any product-specific privacy notice, or for as long as you have an account with us. We may also retain personal data if required by law, or, where applicable, for our legitimate interests, such as abuse detection and prevention, and defending ourselves from legal claims. Residual copies of personal data may be stored in backup systems for a limited period as a security measure to protect against data loss.

8. Your Rights with Your Personal Data

If you live in the European Economic Area ("EEA"), you may have certain legal rights in relation to your personal data that we maintain. Subject to exceptions and limitations provided by applicable law, these may include the right to:

1. be informed of the source(s) from which we obtained your personal data;

2. access and receive a copy of your personal data that we hold;

3. request that we provide you with the categories of personal data we collect, disclose or sell about you (including the categories of sources of such information, the business or commercial purpose for collecting or selling your personal data, and the categories of third parties with whom we share your personal data, all of which we may include in this Privacy Policy);

4. update or correct your personal data if it changes or if you believe that any information that we have collected about you is inaccurate or out-of-date;

5. request information about data processing;

6. to object to or restrict our use or processing of your personal data;

7. be informed of and submit observations regarding automated decision-making;

8. unsubscribe from our email list by clicking the link at the bottom of our marketing emails;

9. unsubscribe from SMS marketing by replying with the word 'Stop';

10. tailoring advertising through third party cookies (refer to our list of advertising services using cookies here for company-specific choices, or visit http://optout.aboutads.info to exercise an industry-wide opt-out);

11. reject or delete cookies through your browser settings;

12. request that we delete or erase your personal data;

13. port your data to another service provider;

14. withdraw your consent in circumstances we rely upon your consent to process your personal data (please note that withdrawing your consent will not affect the lawfulness of any processing we conducted prior to your withdrawal, nor will it affect processing of your personal data conducted upon other lawful processing grounds); or

15. lodge a complaint with a data protection authority.

Please note your rights and choices vary depending upon your location, and some information may be exempt from certain requests under applicable law.

You may be able to exercise some of these rights by submitting your request at https://preferences.aura.com/privacy or by using the settings and tools provided in our services. For example, you may be able to update your user account details via the relevant account settings screen of our apps. You may also be able to opt out from receiving marketing communications from us by clicking an “opt out” or “unsubscribe” link in such communications.

Specifically, the GDPR gives users residing in the EU the rights to access, correct, delete or object to the processing of your personal data. If you are a resident of any EU member country, you may also exercise these rights by submitting your request at https://preferences.aura.com/privacy. For other inquiries, including access and rectification requests, you can contact our Data Protection Officer (DPO). Aura has appointed Bird & Bird DPO Services SRL as a DPO. You can reach Bird & Bird by:

1. emailing DPO.Aura@twobirds.com; or

2. mail at the following address: Bird & Bird DPO Services SRL, Avenue Louise 235 b 1, 1050 Brussels, Belgium.

You may also submit your request at https://preferences.aura.com/privacy or by dialing 1 (844) 914-2991. As permitted by law, we may ask you to verify your identity before taking further action on your request.

9. Legal Basis for Our Processing of Your Personal Data

We may only process personal data where we have a sufficient legal basis, such as your consent or where authorized by law.

Aura Processing Activity	Legal basis under GDPR	Legal basis under Canadian Law
Placement of cookies on Our Site	Consent	Consent

Collecting, processing, and sharing your child’s personal data	Consent*	Consent*
Collection and Processing of your personal data to maintain and fulfill the Services	Contract	Consent
Processing your personal data to improve our Services	Legitimate Interest	Consent
Using your personal data to send you email communications and updates	Legitimate Interest	Consent
Processing your personal data for market research	Legitimate Interest	Consent
Processing your personal data necessary for compliance with a legal or regulatory obligation that we are subject to	Legal Obligation	Authorized By Law

*You expressly authorize and consent to the collection, processing, and sharing of your child’s personal data by adding them to your Family Plan, setting up and by registering their devices with the Parental Control Services and/or creating their profile for the Parental Control Services with us under your account or the Family Plan.

10. Your State Privacy Rights (US Only)

Certain U.S. states may grant you enhanced privacy rights related to our processing of your personal information. Above, we have provided all of the categories of personal information that we collect, process, and disclose to service providers (or ‘processors’). We have also described the various privacy choices you have with respect to your right to know the specific pieces of personal information we process on your behalf, the capability to opt-out of various marketing communications, and the ability to modify or delete your personal information.

In addition to the rights available to all Aura users, you may also have the right to opt-out of the ‘sale’ of your personal information, or the ‘sharing’ of your personal information for targeted or cross-contextual behavioral advertising purposes. Aura does not ‘sell’ personal information as that term is commonly understood, but we enable third party advertising services to utilize pixel tags and cookies on our website or through other tracking technologies in order for Aura to advertise our products and services on other websites or mobile applications. These third party advertising services are considered to be categorized as a ‘sale’ or ‘share’ for targeted or cross-contextual behavioral advertising. You can learn more about this in our Cookie Policy. You can opt out of these ‘sale’ and ‘sharing’ activities by accessing our Your Privacy Choices page located from the footer of our website. This page also describes how we honor opt-out preference signals (aka; ‘Global Privacy Control’) which you may configure through your browser. We do not respond to what is formerly known as ‘Do Not Track’ signals.

In addition, you may have additional rights to limit the use of your ‘sensitive personal information’, which may include government-issued identifiers, precise geo-location

information, or financial account information. For sensitive personal information that we collect, we will only use or disclose it either with your specific consent when required, or as otherwise permitted by law. As we do not use or disclose sensitive personal information for other purposes without your consent, we do not offer you an option to limit the use of sensitive personal information other than through the settings and controls available to you through our software and services, your third party account providers or your browser or mobile device operating system.

Finally, some states have specific rights related to the use of personal information for 'profiling' purposes. Aura does not use your personal information to create profiles about you that we use to inform decisions that have legal or similarly significant effects. However, some of our services such as our financial alerts or Parental Controls software may provide you with information which you may use to make your own decisions. You can control our use of this information for material alerts at any time through your account settings or controls.

You have the right to not be discriminated against. We do not discriminate against you if you exercise any rights provided to you by state law.

Some of our software and services enable parents or guardians to register or otherwise enable Aura to process the personal information associated with children under the age of 16. We do not knowingly sell or share personal information related to children under 16 years of age.

Some states may provide you with the right to appoint an authorized agent to act on your behalf.

Where acceptable by law, we may require you to verify your identity using the processes we describe in the dashboard or the privacy choices page before we fulfill your request, even if you utilize an authorized agent to complete such a request on your behalf.

Some states allow you to appeal a denial of your request to exercise the privacy rights provided by the state law. If you are a resident of Colorado, Connecticut, Oregon, Texas, or Virginia and we deny your request, you can appeal the denial of a request to exercise privacy rights by following the instructions listed in the denial communication, by submitting a request through a privacy inquiry form.

11. Technology Licensing

Aura occasionally licenses its technology to third party partners who may integrate it with applications developed and offered by those partners. Our partners, and not Aura, are responsible for those applications and for determining what data is collected by those applications and how it is processed. Please contact the relevant partner and refer to their Privacy Policy to learn more about how those applications process your personal data.

12. Age Restrictions

The administration, configuration and management of Aura accounts are not intended for and may not be used by minors. In this context, minors are individuals under the age of 18 or as defined by applicable law. Aura does not knowingly collect personal data from minors when creating or administering our accounts or allow them to be an administrator of our services except in certain cases. Minors may use certain of our services but only with the consent and administration of their parent or legal guardian.

In the case of Parental Controls ("Parental Control Services") offered as part of the Aura subscription, our Parental Control Services are expressly designed for parents to monitor the Internet and mobile activity of their children. As a result, certain personal data related to a minor child may be accessible by the parent-administrators of the Parental Control Services, as well as Aura in our administration of the Services. For more information please see the Supplemental Parental Controls Privacy Notice.

If we discover that we have collected personal data from a minor without appropriate consents, we may delete such data without notice. For certain regions, parental consent may be required for processing the personal data of children under the age of 16. In such cases, those under the age of 16 may not use the services without the consent or authorization of their parent or legal guardian. If you believe your minor child has provided personal data without parental or guardian consent, you, as the parent or guardian may contact us by emailing us at privacy@aura.com.

13. Privacy Policy Updates

We may revise this policy from time to time and we will notify you of any changes. Review it occasionally so that you keep up-to-date on our most current practices. We will put the "Last Updated" date at the top of each policy. If you disagree with such an update to this policy, you may cancel your account. If you do not cancel your account before the date the update becomes effective, your continued use of our services will be subject to the updated Privacy Policy.

14. Contact Us

We expect this Privacy Policy to evolve over time and welcome feedback from our users about our privacy practices. If you have any questions or complaints about our privacy practices, you can contact our privacy officer at privacy@aura.com or at the following addresses, based on the product that you use:

Aura Sub LLC dba Aura
250 Northern Ave.
3rd Floor
Boston, MA 02210

Archived Versions

Current version, October 3, 2024: Added supplemental details about Aura Parental Controls andUS State privacy rights; Revised for readability

April 8, 2024: Updated to reflect new capabilities of the Aura service

January 31, 2024: Updates to Terms of Service and Privacy Policy to reflect new regions of the Aura services

September 1, 2023: Reflected new capabilities of the Aura services

August 22, 2023: Reorganized provisions for better readability

April 28, 2023: Correcting the description of our products

March 4, 2023: Added new features such as Call Assistant

July 10, 2022: Clarifying our brands and affiliates, adding in Parental Controls

April 1, 2022: Updated what information we collect and how we share it

November 1, 2021: Rebranding Figleaf to Aura Privacy

June 8, 2021

September 8, 2020

Aura License and Terms of Service (available at: https://www.aura.com/legal/service-terms)

Aura License and Terms of Service

Last Updated:
October 9, 2024

English
—
Français

Part 1. Automatic Renewals And BillingPart 2. General Legal TermsPart 3. Service-Specific TermsPart 4. Limited LicensePart 5. Additional Terms for Residents of CanadaArchived Versions

Effective Date: November 15, 2024

IMPORTANT: PLEASE READ THE FOLLOWING TERMS AND CONDITIONS CAREFULLY BEFORE USING THE SERVICES WE PROVIDE (AS DEFINED BELOW), AS THESE TERMS OF USE CONSTITUTE A BINDING CONTRACT BETWEEN US.

Thanks for choosing Aura. We're here to create a safer internet by making comprehensive digital security simple to understand and easy to use. For the purposes of this License and Terms of Service (collectively "Terms") "Aura", "We" or "Us" means Aura Sub, LLC.

To make it easier to understand, we've divided our legal terms into five parts: (1) Automatic Renewals and Billing, (2) General Legal Terms, (3) Service-Specific Terms, (4) License Terms, (5) Additional Terms for Residents of Canada.

These Terms represent a binding contract between you and Aura. These Terms apply to your use of any products or services offered by Aura as described in these Terms (collectively, the "Services"), including all software provided as a part of our Services (the "Software"), whether through the website found at https://www.aura.com/ or a mobile application, or in any other manner.

You agree to be bound by these Terms when you sign up for or use the Services, download or use any Software in connection with the Services, and any time you access or use the Services. **We encourage you to read our Terms carefully. If you do not agree to be bound by these terms, you should not use the Services.**

IN PARTICULAR, THESE TERMS CONTAIN AN ARBITRATION AGREEMENT THAT REQUIRES U.S. USERS TO RESOLVE DISPUTES WITH AURA OR ITS AFFILIATES THROUGH ARBITRATION ON AN INDIVIDUAL BASIS. ARBITRATION ON AN INDIVIDUAL BASIS MEANS THAT YOU WAIVE THE RIGHT FOR A JUDGE OR JURY TO DECIDE YOUR CLAIMS, AND THAT YOU MAY NOT PROCEED IN A CLASS, CONSOLIDATED, OR REPRESENTATIVE CAPACITY. SEE SECTION 2.12 BELOW FOR THE DETAILS OF THE ARBITRATION AGREEMENT.

Part 1. Automatic Renewals And Billing

This section does not apply to you if you are an employee who is provided the Services via an arrangement your employer has with us under Section 1.7(b) (see below).

1.1 Subscription and Enrollment. Depending upon the Services you subscribe or register to use, you may be permitted to be enrolled as follows: (1) by telephone, (2) by the Aura website, (3) by another method permitted by Aura or (4) by a third-party who is authorized on Aura's behalf or provide your personal information to enroll you on your behalf. Our Services are generally billed on a subscription basis ("Subscription"). If applicable and you elect to enroll in a Subscription, you will be billed in advance for your Subscription on a recurring and periodic basis ("Billing Cycle"). The length of your Billing Cycle will depend on the Subscription you select. Billing Cycles will typically consist of

one (1) month or one (1) year terms ("Subscription Term"). You agree to pay for the Subscription that you select. You also authorize us to automatically charge the payment method our service provider has on file based on the Billing Cycle applicable to your Subscription. If you have a yearly subscription, upon renewal, we may automatically change the payment cycle of your subscription from a yearly payment cycle to a monthly payment cycle provided we give you at least ten (10) days' prior notice of such change and the total fees owed for one year of monthly payments equals your yearly subscription price.

1.2 Automatic Renewals and Cancellation. At the end of each Billing Cycle, your Subscription will automatically renew for an additional Billing Cycle at the price shown in your account dashboard or otherwise communicated to you by us pursuant to Section 1.6 ("Renewal Price") unless you or we have canceled your Subscription at least one (1) day prior to the commencement of your next Billing Cycle or in accordance with Section 1.4 below. We will email you in advance to let you know your Subscription is due for renewal and provide the renewal price in your account dashboard. You may cancel your Subscription by contacting us via the phone number in your account dashboard or by logging into your account. Once we or you have canceled your Subscription, your recurring subscription fees for the Services will no longer be charged to the payment method our service provider has on file for your account, and your Subscription will remain active only until the end of the current Billing Cycle. Aura is under no obligation to provide you any refund if you cancel your Subscription early.

For individuals residing in the province of Quebec, please see additional provisions at Section 5.1.2 for automatic renewals.

1.3 Plan Switching. In the event we allow you to switch your Aura Subscription plan, we will send you an email confirming the plan change, and reflect any Service Credits (as defined in Section 1.8) or amount owed due to the difference in changing plans, and the Billing Cycle for the new plan. Your plan switch will be effective as of the date shown in your account profile and confirmation email, which will generally be tied to the date you elect to switch your plan.

1.4 Money Back Guarantee. Certain Services may include a Money Back Guarantee. If a Money Back Guarantee is offered for the Services, the terms of the Money Back Guarantee will be visible in your account profile.

1.5 Payment Methods. A valid payment method is required to process the payment for your Subscription. You shall provide us or our third-party payment processor with accurate and complete billing information which may include full name, address, state/province/territory, zip/postal code, and valid payment method information. By submitting such payment information, you automatically authorize us to charge all Subscription fees incurred through your account to any such payment instruments.

Unless we expressly provide otherwise, all amounts paid are non-refundable. You further agree to be responsible for any applicable state/provincial/territorial, federal or

other taxes that may be associated with the Services, including sales taxes, along with any transaction fees and currency conversions added by your financial institution and intermediaries. All amounts are in U.S. Dollars for U.S. customers and Canadian Dollars for Canadian Customers.

If the billing method you provide is a credit or debit card ("Payment Card"), you:

(a) represent that you are authorized to use such Payment Card;

(b) authorize us to charge your Payment Card periodically for the Subscription fees when due, including upon sign up for the Services and each subsequent Billing Cycle;

(c) agree to keep your Payment Card details valid and current; and

(d) agree to pay any processing fees that are charged by the third-party payment processors or Payment Card issuer.

If your Payment Card is declined when we attempt to charge it, we may try to charge it again at a later time. If we do not receive payment, we may suspend or terminate your Subscription.

1.6 Fee Changes. We may change the fees that we charge for the Services at any time at our sole discretion, provided that we will give you at least thirty (30) days of prior notice of such changes. Unless otherwise specified in such notice to you, any changes to the Subscription fees will take effect in the next Billing Cycle immediately following our notice to you.

1.7 Cancellations with Aura Authorized Resellers. If you enrolled in our Services as part of an employee benefits program or Reseller Program, the following terms apply.

1. Employee Benefits Voluntary User Program. If you enrolled in our Services via an Employee Benefits Voluntary User Program (where you are offered to sign up and pay for the Services via payroll deduction), you must contact your employer to cancel the Service. The disposition of any refunds is between you and your employer.
2. Employee Benefits Employer Paid Program. If you enrolled in our Services via an Employee Benefits Employer Paid Program (where the employer pays for the Services to be provided with an option for you to purchase additional Services via credit card purchase), any Services paid for by the employer will not result in any refund to you at time of cancellation. If you purchased additional Services via credit card, no refunds are provided. The Services will continue to be accessible until the last day of your current month's billing cycle.
3. Employee Benefits User Direct Enrollment Program. If you enrolled in our Services via an Employee Benefits User Direct Enrollment Program (where you pay for the Services directly), no refunds are provided. The Services will continue to be accessible until the last day of your current month's billing cycle.
4. Other Reseller Programs. If you enrolled in our Services via a reseller program ("Reseller Program"), no refunds are provided by Aura. The Services will continue to be accessible until the last day of your current month's billing cycle. Should you have

questions, please contact your Reseller as any disposition of refunds, if owed, is between you and the Reseller.

1.8 Service Credits. At any time, and for any reason, we may provide you with credits that are redeemable for our Services ("Service Credits"). Unless otherwise required by applicable law, Service Credits are not legal tender or currency; are not redeemable, refundable, or exchangeable for any sum of money or monetary value; have no equivalent value in fiat currency; do not act as a substitute for fiat currency; and do not constitute or confer upon you any personal property right. Service Credits are non-transferable and may be used only in connection with the Services. **Service Credits are not refundable and expire one year after the date of purchase or issuance if not used, unless otherwise specified at the time of purchase.**

Part 2. General Legal Terms

2.1 Access and Eligibility. To use Our Services, you must be Eligible, establish a user account, and enroll in a Subscription to the Services. In order to access and use certain Services, you may be required to download and install certain Software on a device. "Eligible" means that you reside in either Canada or the United States of America and (i) you are 18 years of age or above the age of majority in your jurisdiction of residence (whichever is greater); or (ii) you are 13 years of age or older (in Quebec, 14 years of age or older), but under the age of majority in your jurisdiction of residence and are using the Services with the permission and involvement of your parent or legal guardian who has agreed to these Terms. By downloading, using, or accessing the Services, you represent and warrant that you are Eligible. Under no circumstances may anyone under 13 years of age use or access the Services at any time or in any manner or submit any information through the Services outside of the circumstances outlined in this section. Our Services are not available to persons who are not Eligible or to any users previously suspended, terminated, or removed from the Services by Aura.

If Aura does not receive all the required personal information (which is necessary to provide the Services) during the enrollment process to provide the features in the Subscription plan you selected, you agree that we may use our databases, or other resources to attempt to complete the required information on your behalf. If you do not provide this necessary information and we cannot verify your identity, our service providers may be unable to successfully provide the features in the Subscription plan you've selected. As such, we may refuse to allow you to use certain features (for example, some credit based features) and may automatically enroll you in a version of our Services that has fewer features (for example, only including non-credit features).

2.2 User Data, Accounts and Passwords. You are fully and solely responsible and liable for the content and data you enter into our Services, including your user name and password. You are solely responsible for ensuring that you keep your user name and password safe. You agree not to disclose your password to any third-party and must notify us immediately of any unauthorized use of your account. We are not responsible for your failure to do so, or for any delay in suspending or terminating your

account after you do. You are responsible for all use of your account. We may reject, or require that you change, any user name or password, in our sole discretion.

2.3 User Submissions; Feedback; Beta Features; Updates. If you provide to Aura, either directly or through a third-party such as the Apple App Store, feedback and/or reviews, suggestions, comments, or ideas relating to the Services ("Submissions"), you grant, to the maximum extent permitted by applicable law, Aura and its affiliated companies a royalty-free, perpetual, irrevocable, worldwide, unlimited, nonexclusive license to use, reproduce, create derivative works from, modify, publish, edit, translate, distribute, perform, and display the Submission in any media or medium, or any form, format, or forum, now known or hereafter developed, to improve or market the Services or for any other reasonable business purpose. We may sublicense these rights through multiple tiers of sublicenses. No compensation will be paid with respect to the use of your Submission. Aura is under no obligation to post or use any Submission you may provide and Aura may remove any Submission at any time in its sole discretion and without notice. By providing a Submission to Aura, you represent and warrant that you own or otherwise control all of the rights to your Submission that are necessary for you to provide it, including Intellectual Property Rights. You agree that: (i) all content of your Submissions must be accurate; (ii) you will not provide a Submission that is known by you to be false, inaccurate or misleading and/or may be reasonably considered to be defamatory, libelous, hateful, offensive, unlawfully threatening or unlawfully harassing to anyone; (iii) you will not provide a Submission that infringes or violates a third-party's Intellectual Property Rights or other proprietary rights or rights of publicity or privacy; (iv) you will not provide a Submission that violates any applicable law, statute, ordinance or regulation; (v) you will not provide a Submission for which you were compensated or granted any consideration by any third-party; (vi) you shall not provide any Submission that includes information that references other websites, addresses, email addresses, contact information, phone numbers, or other personally identifiable information for anyone;] (vii) you will not provide a Submission that contains any potentially damaging computer programs or files; and Submissions shall not be deemed confidential and Aura shall not have any obligation to keep any such material confidential.

Also we may, in our sole discretion, include new and/or updated beta features in the Services for your use and which permit you to provide feedback. Using beta features may subject you to the payment of fees. You understand and agree that your use of the beta features is voluntary. The beta features are provided on an "as is" basis and you acknowledge and agree that all use of beta features is at your personal risk. Certain beta features may be subject to additional terms or an agreement.

We may from time to time develop and provide updates for the Services, which may include upgrades, bug fixes, patches and other error corrections and/or new features, functionality, tools or content (collectively, "Updates"). Updates may also modify or delete features, functionality, tools or content in their entirety. If you do not download and install the most recent Updates, portions of the Services may not properly operate. All Updates will be deemed part of the Services and be subject to all terms and conditions of the Terms. You agree that Aura has no obligation to provide any

Updates or to continue to provide or enable any particular features, functionality, tools or content.

2.4 Free Trials. EXCEPT WHERE EXPRESSLY PROHIBITED FOR INDIVIDUALS RESIDING IN THE PROVINCE OF QUEBEC, Aura may, in its sole discretion, offer a Subscription with a free trial for a limited period (“Free Trial”). If we offer you a Free Trial, the specific terms of your Free Trial will be provided at signup and/or in the promotional materials (such as, but not limited to, emails) describing the Free Trial and your use of the Free Trial is subject to your compliance with such specific terms. Free Trials are only available to users who have not subscribed to the Services in connection with a Free Trial being offered in the last 12 months or other duration as clearly defined by Aura in the signup flow and/or in the applicable promotional materials.

You may be required to enter your billing information to sign up for the Free Trial. If you do enter your billing information when signing up for the Free Trial, you will not be charged by us until the Free Trial has expired. You may cancel your Subscription within at least 24 hours prior to the end of your Free Trial through your Aura dashboard and unless you cancel during the aforementioned timeframe. You will be automatically charged the applicable Subscription fees for the type of Subscription you have selected. At any time and without notice, we reserve the right to (i) modify the terms and conditions of the Free Trial offer, or (ii) cancel such Free Trial offer. Before charging you at the end of your free trial period, we will notify you of the applicable fees.

2.5 Links to Third-Party Websites and Products. The Services may also provide hyperlinks to third-party websites, resources, or services (“Third-Party Content”). You acknowledge and agree that Aura is not responsible or liable for (i) the availability, terms or practices of such websites, resources or services, or (ii) the content, products or services available on or through such websites, resources or services, including that any information provided is complete, accurate or up-to-date. Links to such websites, resources or services do not imply any endorsement by Aura of such websites, resources or services or the content, products or services available on or through such websites, resources or services. If you choose to access or use any Third-Party Content, your personal information may be available to a third-party content provider. These Terms will not apply to your activities or any information you disclose while using third-party products or services or otherwise interacting with third parties. How third parties handle and use your personal information related to their sites and services is governed by their security, privacy, and other policies, if any, and not our policies. Under no circumstances shall we be held responsible or liable, directly or indirectly, for any loss or damage caused or alleged to have been caused to a user in connection with the use of or reliance on any Third-Party Content.

2.6 Data from Third-Party Services and Products. Some features and functionalities may enable you to share Your’s or a Family Member’s data with a Third-Party service or product (“Third-Party Service Data”) with Aura or enable Aura to access said Third-Party Service Data. By sharing or enabling access to Third-Party Service Data, you represent and warrant that you own this information and have the right to provide it to us

or enable our access to it. You acknowledge and agree that you, not Aura, are responsible and liable for compliance with any terms or conditions associated with the Third-Party Service Data (“Third-Party Service Terms”) and under no circumstances shall we be held responsible or liable, directly or indirectly, for any loss or damage caused or alleged to have been caused due to your failure to comply with any Third-Party Service Terms.

2.7 Indemnification. To the fullest extent permitted by law, you agree to indemnify and hold Aura, its affiliates, agents, suppliers, vendors, contractors, resellers, third-party partners, and licensors, and each of their respective contractors, subcontractors, officers, directors, shareholders, employees, agents, and its third-party suppliers, licensors, and partners (collectively, the “Aura Entities”) harmless from any claims, losses, damages, liabilities, including legal fees and expenses, arising out of your access to or use of the Services. Aura reserves the right, at your expense, to assume the exclusive defense and control of any matter for which you are required to indemnify the Aura Entities, and you agree to cooperate with Aura’s defense of these claims. Aura will use reasonable efforts to notify you of any such claim, action, or proceeding upon becoming aware of it.

2.8 Export. The Services, or certain portions thereof, may be subject to United States export controls. You may not export or re-export any aspect of the Services without (a) the prior written consent of Aura, (b) complying with any applicable export control laws, and (c) obtaining all appropriate permits and licenses. In any event, you may not remove or export from the United States or allow the export or re-export of any part of the Services in violation of any restrictions, laws, or regulations of the United States Department of Commerce, the United States Department of Treasury Office of Foreign Assets Control, or any other United States or foreign agency or authority. The Services may contain information that is controlled and restricted from export by the United States export controls restrictions, regulations, and laws described above (the “Controlled Information”). If Aura, in its sole discretion, determines that it cannot implement the Services in a manner to exclude access to Controlled Information where required, if you are in a country or territory that is subject to such regulation, you shall not be provided access to the Services.

2.9 Disclaimers; No Warranties. TO THE FULLEST EXTENT PERMITTED UNDER LAW, SAVE FOR THE RIGHTS GRANTED TO CONSUMERS UNDER APPLICABLE LAWS, AND EXCEPT WHERE EXPRESSLY PROHIBITED BY LAW FOR CONSUMERS RESIDING IN THE PROVINCE OF QUEBEC, Aura disclaims all warranties, statutory, express, or implied, including, but not limited to, implied warranties of merchantability, fitness for a particular purpose, and non-infringement of proprietary rights. No information, whether oral or written, obtained by you from Aura or through the Services will create any warranty not expressly stated herein. You expressly agree that the use of the Services is at your sole risk. The Services and any data, information, third-party software, services, or applications made available in conjunction with or through the Services are provided on an “as is” and “as available”, “with all faults” basis and with no assurances that the Services will withstand attempts to evade security

mechanisms or that there will be no cracks, bugs, disablements or other circumvention. Aura does not warrant that the Services will be uninterrupted or free of errors, viruses, or other harmful components and does not warrant that any of the foregoing will be corrected. You understand and agree that if you use, access, or download the Services, or otherwise obtain or transmit materials, data, or other content while using the Services, you do so at your discretion and risk. When using the Services, information will be transmitted over a medium that is beyond the control and jurisdiction of Aura, its partners, advertisers, and sponsors, or any other third-party mentioned on the Services. Accordingly, Aura assumes no liability for or relating to the delay, failure, interruption, or corruption of any data or other information transmitted in connection with the use of the Services.

SOME JURISDICTIONS DO NOT ALLOW THE EXCLUSION OF IMPLIED WARRANTIES OR CERTAIN DISCLAIMERS, SO SOME OF THE ABOVE EXCLUSIONS AND TERMS MAY NOT APPLY TO YOU. CONSUMER RIGHTS MAY VARY FROM ONE JURISDICTION TO ANOTHER JURISDICTION. TO THE EXTENT YOU MAY HAVE CERTAIN RIGHTS UNDER APPLICABLE LAWS IN YOUR JURISDICTION, NOTHING IN THESE TERMS IS INTENDED TO AFFECT THOSE RIGHTS, IF THEY APPLY.

2.10 Limitation of Liability and Damages. TO THE FULLEST EXTENT PERMITTED UNDER LAW AND EXCEPT WHERE EXPRESSLY PROHIBITED FOR INDIVIDUALS RESIDING IN THE PROVINCE OF QUEBEC, AURA AND THE AURA ENTITIES WILL HAVE NO OBLIGATION OR LIABILITY (WHETHER ARISING IN CONTRACT, WARRANTY, TORT, INCLUDING NEGLIGENCE, PRODUCT LIABILITY, OR OTHERWISE) FOR ANY DAMAGES OR LIABILITIES, INCLUDING DIRECT, INCIDENTAL, INDIRECT, SPECIAL, OR CONSEQUENTIAL (INCLUDING ANY LOSS OF DATA, REVENUE OR PROFIT OR DAMAGES ARISING FROM PERSONAL INJURY/WRONGFUL DEATH) ARISING CONCERNING YOUR USE OF THE SERVICES, EVEN IF WE HAVE BEEN ADVISED OF THE POSSIBILITY OF SUCH DAMAGES. THIS LIMITATION APPLIES TO DAMAGES ARISING FROM (i) USE OF OR INABILITY TO USE THE SERVICES, (ii) COST OF PROCUREMENT OF SUBSTITUTE GOODS AND SERVICES, (iii) UNAUTHORIZED ACCESS TO OR ALTERATION OF YOUR TRANSMISSIONS BY THIRD PARTIES, (iv) THIRD-PARTY CONTENT MADE AVAILABLE TO YOU THROUGH THE SERVICES, OR (v) ANY OTHER MATTER RELATING TO THE SERVICES. THE EXCLUSIONS AND LIMITATIONS OF DAMAGES SET FORTH ABOVE ARE FUNDAMENTAL ELEMENTS OF THE BASIS OF THE BARGAIN BETWEEN AURA AND YOU.

TO THE FULLEST EXTENT PERMITTED UNDER LAW, NEITHER AURA, CSID, EQUIFAX, TRANSUNION, EXPERIAN NOR ANY OF THEIR RESPECTIVE AFFILIATES SHALL HAVE ANY LIABILITY TO YOU AS AN AGENT IN OBTAINING COPIES OF ANY OF YOUR CREDIT REPORT, CREDIT ALERT REPORT, QUARTERLY UPDATE, OR CREDIT SCORE. NEITHER AURA, CSID, EQUIFAX, TRANSUNION, EXPERIAN NOR ANY OF THEIR RESPECTIVE AFFILIATES OR CREDIT INFORMATION SUBCONTRACTORS MAKE ANY WARRANTY, EXPRESS

OR IMPLIED, FOR THE ACCURACY OF THE INFORMATION CONTAINED IN OR PROVIDED IN CONJUNCTION WITH THE AURA SERVICES. NEITHER AURA, CSID, EQUIFAX, TRANSUNION, EXPERIAN NOR ANY OF THEIR RESPECTIVE AFFILIATES ASSUME ANY LIABILITY FOR DAMAGES, DIRECT OR INDIRECT, CONSEQUENTIAL OR INCIDENTAL, IN CONNECTION WITH THE PERFORMANCE OF THE SERVICES OR YOUR REQUEST, USE, OR ATTEMPTED USE OF THE SERVICES. NEITHER AURA NOR ANY OF THEIR RESPECTIVE AFFILIATES OR CREDIT INFORMATION SUBCONTRACTORS ARE RESPONSIBLE FOR NEGATIVE FACTUAL INFORMATION CONTAINED IN ANY REPORTS YOU RECEIVE AS PART OF THE SERVICE. THE AGGREGATE LIABILITY OF ALL SUCH PARTIES TO YOU, IN ANY EVENT, IS LIMITED TO THE AMOUNT WHICH YOU HAVE PAID AURA FOR YOUR MEMBERSHIP. AURA PRODUCTS AND SERVICES ARE NOT A CREDIT COUNSELING SERVICE AND DO NOT PROMISE TO HELP YOU OBTAIN A LOAN OR IMPROVE YOUR CREDIT RECORD, HISTORY, OR RATING. THE TERMS OF THIS SECTION SHALL SURVIVE ANY TERMINATION, CANCELLATION, OR EXPIRATION OF THIS AGREEMENT.

WE ACKNOWLEDGE CERTAIN JURISDICTIONS DO NOT ALLOW LIMITATION OR EXCLUSION OF LIABILITY FOR INCIDENTAL OR CONSEQUENTIAL DAMAGES, AND AGREE THAT, TO THE EXTENT YOU HAVE ADDITIONAL RIGHTS UNDER APPLICABLE LAWS OF YOUR JURISDICTION, CERTAIN OF THESE TERMS MAY NOT APPLY TO YOU AND THESE TERMS ARE NOT INTENDED TO AFFECT YOUR APPLICABLE RIGHTS.

2.11 Government Use. This Section 2.11 applies where the Services are provided for the benefit of a U.S. governmental end-user. As defined in FAR section 2.101, the Services are “commercial items” and according to FAR 12.212 and DFARS section 252.227-7014(a)(1) and (5) are deemed to be “commercial computer software” and “commercial computer software documentation.” Consistent with FAR section 12.212 and DFARS section 227.7202, any use modification, reproduction, release, performance, display, or disclosure of such commercial software or commercial software documentation by the U.S. Government will be governed solely by these Terms and will be prohibited except to the extent expressly permitted by these Terms.

2.12 Dispute Resolution By Binding Individual Arbitration. Most customer concerns can be resolved quickly and to the customer's satisfaction by contacting us. If customer service is not able to resolve your concern or complaint to your satisfaction, you agree to give Us a fair opportunity to resolve any complaint, claim or dispute you may have informally and shall provide Us with a written notice of your claim in the manner described in the Notices; Contact Us section, so that we can cooperate with each other to try to address the matter amicably prior to engaging in any formal dispute resolution proceedings.

In the unlikely event that customer service is unable to resolve a complaint you may have with Aura or Aura’s service provider(s) to your satisfaction (or if Aura and/or Aura’s service provider(s) have not been able to resolve a dispute with you after attempting to

do so informally), you, on the one hand, and Aura and/or Aura's service provider(s), on the other, each agree to resolve those disputes under the Arbitration Agreement contained in this Section 2.12. The Arbitration Agreement requires you to resolve all disputes (other than those expressly exempted in this Section 2.12) through binding arbitration on an individual basis, instead of in courts of general jurisdiction. Arbitration is more informal than a lawsuit in court. Arbitration uses a neutral arbitrator instead of a judge or jury, allows for more limited discovery than in court, and is subject to very limited review by courts. Arbitrators can award the same damages and relief that a court can award. Any arbitration under these Terms will take place on an individual basis; class arbitrations and class actions are not permitted. However, in arbitration, you, on the one hand, and Aura and/or Aura's service provider(s), on the other, will be entitled to recover attorneys' fees to the same extent they would be available in court.

Arbitration Agreement

1. Mandatory Arbitration of Disputes. We each agree that any dispute, claim or controversy arising out of or relating to these Terms or the breach, termination, enforcement, interpretation or validity thereof or the use of the Services (collectively, "**Disputes**") will be resolved **solely by binding, individual arbitration and not in a class, representative or consolidated action or proceeding**. You and Aura agree that the U.S. Federal Arbitration Act governs the interpretation and enforcement of these Terms, and that you and Aura are each waiving the right to a trial by jury and the right to participate in a class action. The parties further agree that, in order to bring a claim in arbitration, they must first be able to satisfy the federal standing requirement under Article III of the United States Constitution with respect to their claim, and that the arbitrator shall be empowered to make a binding determination on standing. This arbitration provision shall survive termination of these Terms.
2. Exceptions. As limited exceptions to Section 2.12(a) above: (i) we both may seek to resolve a Dispute in small claims court if it qualifies; (ii) we each retain the right to seek injunctive or other equitable relief from a court to prevent (or enjoin) the infringement or misappropriation of our intellectual property rights (iii) claims you may have which relate to your credit report, or any claims arising out of or relating to the Fair Credit Reporting Act ("FCRA") and/or the FCRA's law equivalent(s) in other jurisdictions are not subject to this Arbitration Agreement.
3. Conducting Arbitration and Arbitration Rules. The arbitration will be conducted by the American Arbitration Association ("**AAA**") under its Consumer Arbitration Rules (the "**AAA Rules**") then in effect, except as modified by these Terms. The AAA Rules are available at www.adr.org or by calling 1-800-778-7879. A party who wishes to start arbitration must submit a written Demand for Arbitration to AAA and give notice to the other party as specified in the AAA Rules. The AAA provides a form Demand for Arbitration at www.adr.org.
4. Any arbitration hearings will take place in the county (or parish) where you live, unless we both agree to a different location. The parties agree that the arbitrator shall have exclusive authority to decide all issues relating to the interpretation, applicability, enforceability and scope of this arbitration agreement.
5. Arbitration Costs. Payment of all filing, administration and arbitrator fees will be governed by the AAA Rules, and we won't seek to recover the administration and arbitrator fees we are responsible for paying, unless the arbitrator finds your Dispute

frivolous. Both You and Aura will be entitled to recover attorneys' fees to the same extent they would be available in court.

6. Injunctive and Declaratory Relief. Except as provided in Section 2.12(b) above, the arbitrator shall determine all issues of liability on the merits of any claim asserted by either party and may award declaratory or injunctive relief only in favor of the individual party seeking relief and only to the extent necessary to provide relief warranted by that party's individual claim. To the extent that you or we prevail on a claim and seek public injunctive relief (that is, injunctive relief that has the primary purpose and effect of prohibiting unlawful acts that threaten future injury to the public), the entitlement to and extent of such relief must be litigated in a civil court of competent jurisdiction and not in arbitration. The parties agree that litigation of any issues of public injunctive relief shall be stayed pending the outcome of the merits of any individual claims in arbitration.
7. Class Action Waiver. **YOU AND** Aura **AGREE THAT EACH MAY BRING CLAIMS AGAINST THE OTHER ONLY IN YOUR OR ITS INDIVIDUAL CAPACITY, AND NOT AS A PLAINTIFF OR CLASS MEMBER IN ANY PURPORTED CLASS OR REPRESENTATIVE PROCEEDING.** Further, if the parties' Dispute is resolved through arbitration, the arbitrator may not consolidate another person's claims with your claims and may not otherwise preside over any form of a representative or class proceeding. If this specific provision is found to be unenforceable, then the entirety of this Dispute Resolution section shall be null and void.
8. Severability. With the exception of any of the provisions in Section 2.12(f) of these Terms ("**Class Action Waiver**"), if an arbitrator or court of competent jurisdiction decides that any part of these Terms is invalid or unenforceable, the other parts of these Terms will still apply.

2.13 Prohibited Conduct. BY USING THE SERVICES YOU AGREE NOT TO:

- use the Services for any fraudulent, harassing, or abusive purpose, or to damage or cause risk to our business, reputation, employees, subscribers, facilities, or any person;
- use the Services for any illegal purpose, or in violation of any local, state/provincial/territorial, national, or international law;
- use the Services for any commercial use, it being understood that the Services are for personal, non-commercial use only;
- use the Services if you are not Eligible;
- remove, circumvent, disable, damage, or otherwise interfere or deny service in any way or form with security-related features of the Services, features that prevent or restrict use or copying of the Software, or features that enforce limitations on the use of the Services;
- reverse engineer, decompile, disassemble or otherwise attempt to discover the source code of the Services or any part thereof;
- intentionally interfere with or damage the operation of the Services, by any means, including uploading or otherwise disseminating viruses, adware, spyware, worms, or other malicious code.
- Obtain, export, move or copy any data, information or material when you do not have the right to do so.
- Impersonate any person or entity, engage in sender address falsification, forge anyone else's digital or manual signature, or perform any other similar fraudulent activity (for example, "phishing").

- Upload, post, publish, transmit, reproduce, create derivative works of, or distribute in any way information, software or other material, or otherwise that is protected by copyright or other proprietary right, without obtaining any required permission of the owner;
- Upload, post, publish, transmit, reproduce, create derivative works of, or distribute in any way information that is confidential, libelous, defamatory, obscene, pornographic, abusive, indecent, threatening, harassing, hateful, or offensive or otherwise unlawful;

2.14 No Tax, Legal, or Medical Advice; No Credit Repair Services. You acknowledge and agree that we are not providing any medical, legal, tax, or financial advice by providing the Services to you. We are also not a credit repair agency and use of the Services will not repair your credit or improve your creditworthiness.

2.15 Additional Applicable Terms.

1. General. Certain areas, features, or functionality of the Services may be subject to different or additional terms, rules, guidelines, or policies ("Additional Rules") and not every Subscription will include the same features (for example, Subscriptions purchased via an authorized third-party may not include all of the same features as a Subscription purchased directly from Aura). Not all features may be available on all devices. We may provide such Additional Rules to you via postings, pop-up notices, links, or other means at the time that you access or use the relevant area, feature or functionality. From time to time, such Additional Rules may conflict with these Terms. In the event of such a conflict, the Additional Rules will control. Any reference to the "Terms" in this agreement includes the Additional Rules.
2. Additional License Terms for Apple App Store. If the Software is provided to you through Apple Inc. (Apple Inc. together with all of its affiliates, "Apple") App Store, the following terms and conditions apply to you in addition to all the other terms set forth herein: (a) Apple is not responsible for the Services and has no obligation to furnish any maintenance or support services for the Software or the Services; (b) In the event of any failure of the Software to conform to any applicable warranty, you may notify Apple, and Apple will refund the purchase price for the Software (if any) to the Customer. Except for the foregoing, to the maximum extent permitted by applicable law, Apple will have no other warranty obligation whatsoever for the Software, and any other claims, losses, liabilities, damages, costs, or expenses attributable to any failure to conform to any warranty will be governed by the Terms; (c) Any claim in connection with the Software related to product liability, a failure to conform to applicable legal or regulatory requirements, or claims under consumer protection or similar legislation is governed by this Agreement, and Apple is not responsible for such claim. (d) Any third-party claim that the Software or your possession and use of the Software infringe that third-party's Intellectual Property Rights will be governed by this Agreement, and Apple will not be responsible for the investigation, defense, settlement, and discharge of such intellectual property infringement claim; (e) Apple shall be a third-party beneficiary of this Agreement and upon your acceptance of this Agreement, Apple will have the right (and will be deemed to have accepted the right) to enforce this Agreement against you.
3. Additional License Terms for the Google Play Store. If the Software is provided to you through the Google Play Store ("Google-Sourced Software"), then the following **Google Play Store** terms and conditions [link here] apply to you in addition to all the other terms set forth herein: (a) you acknowledge that these Terms for the Services, including the Google-Sourced Software, are between you and Aura only, and not with Google, Inc. ("Google"); (b) your use of Google-Sourced Software must comply with Google's then-

current Google Play Store Terms of Service; (c) Google is only a provider of the Google Play Store where you obtained the Google-Sourced Software; (d) Aura, and not Google, is solely responsible for its Google-Sourced Software; (e) Google has no obligation or liability to you with respect to Google-Sourced Software or these Terms; and (f) you acknowledge and agree that Google is a third-party beneficiary to these Terms as it relates to Aura's Google-Sourced Software.

4. Other Stores. If our Software is downloaded from any other store, platform, marketplace (e.g., Microsoft Store), you acknowledge that you have read, understood, and agree to the customer terms of use of such stores, platforms, and marketplace. Aura is the licensor of the Software and the provider of the Services and any third party (e.g., operator of the store, platform, marketplace) is not a party to these Terms.

2.16 Privacy. We respect your privacy and have taken specific steps to help protect it. Your submission of personal information through the Services is governed by our Privacy Policy. By participating in the Services, you acknowledge that you have reviewed and understand our Privacy Policy and consent to the practices described in that policy.

2.17 Cancellation

1. Cancellation by Aura. Aura may terminate your use of the Services or discontinue providing access to the Services at any time and for any reason, including, but not limited to any actual or suspected breach by you of these Terms or any other unacceptable or objectionable use of the Services, as determined by Aura in its sole discretion. You agree that any termination of your access to the Services may be effected without prior notice and you agree that Aura will not be liable to you or any third-party for any such termination. All refunds are issued at Aura's sole discretion and any refund request may be denied for any or no reason.
2. Cancellation by You. You may cancel your Subscription at any time, but please note that such cancellation will be effective at the end of your then-current Subscription period.
3. Termination of this Agreement. Upon cancellation or termination of the Services by either You or Aura, this Agreement shall terminate except as set forth in Section 2.23.

2.18 Force Majeure. Due to the nature of the Services, actual coverage, speeds, server locations, and quality of Services may vary. We aim to improve and provide Services at all times, but operation of the Services are dependent on the internet and third party internet connections, equipment or infrastructure, as well as third party service providers. From to time to time, Services may be not available without a prior notice or Our liability, including when (a) We test, update, expand, add, or remove our Services, features, functionalities, including those required to reflect changes in relevant laws and regulatory requirements (b) we experience temporary interruptions due to technical difficulties, maintenance or human errors; or (b) Force Majeure Event (as defined below) cause interruption to the Services.

Neither party will be responsible or have any liability for any delay or failure to perform, or inadequacy in performance, to the extent caused by unforeseen circumstances or causes beyond a party's reasonable control ("Force Majeure Event"), which may include

natural disaster (such as earthquake, fire, flood, severe weather, or pandemic), sanctions, embargoes, strikes, labor disturbances, civil unrest or riots, unavailability or delay of suppliers or licensors, riots, acts of terrorism or criminal activity, war, failure or interruption of the internet or related infrastructure, power failures, acts or orders of civil and government authorities, or any other act of God; provided that each party will use reasonable efforts to limit the resulting delay or failure in its performance and the foregoing shall not alleviate any applicable payment obligations.

2.19 Trademark and Copyright. © 2024 Aura All rights reserved. "AURA," "IDENTITY GUARD" (and combinations thereof) are trademarks and/or registered trademarks of Aura. The following are trademarks of third parties: Equifax is a registered trademark of Equifax, Inc.; Experian is a registered trademark of Experian, Inc.; TransUnion is a registered trademark of TransUnion, LLC; and other trademarks are trademarks of their respective owners.

All aspects of the Services and their content, features, and functionality are owned by Aura, our licensors, or other content suppliers, and are protected by copyright and other intellectual property laws. See Part IV of these Terms for the details of the limited license under which you are permitted to use the Services.

2.20 Account Data and Usage Data.

1. Account Data and Licenses. You hereby grant Aura a worldwide, revocable, non-exclusive, royalty-free, sublicensable, and transferable license and all other rights required or necessary to reproduce, distribute, publicly display, publicly perform, create derivative works of, and otherwise use, modify, and exploit Your Account Data for the following purposes ("Permitted Uses"): (i) consistent with this Agreement and as required to perform our obligations and provide the Services; (ii) in accordance with our Privacy Policy; (iii) as authorized or instructed by you; (iv) as required by Law; or (v) for legal, safety or security purposes. "Account Data" means electronic data and information submitted by You to the Services or collected and processed by Aura for You as a result of your use of the, excluding Usage Data. Account Data may include personal data.
2. Usage Data**.** Aura owns all rights, title, and interest in and to telemetry data, product usage data, diagnostic data, and similar data ("Usage Data") that Aura collects or generates in connection with Your use of the Services.

2.21 Notices; Contact Us.

1. Notices. All notices must be in writing and will be deemed given when: (a) personally delivered, (b) verified by a written receipt, if sent by postal mail with verification of receipt service or courier, (c) received, if sent by postal mail without verification of receipt, or (d) verified by automated receipt or electronic logs if sent by email, provided that no bounce or other technical error message was received in response. Notices to Aura must be sent to Aura Sub, LLC at 250 Northern Ave., 3rd Floor, Boston, MA 02210, marked to the attention of the Legal Department, with a copy emailed to legalnotices@aura.com. Email alone is insufficient for providing non-routine legal notices to Aura such as notices related to disputes or arbitration, indemnification claims, breach notices, and termination notices ("Non-Routine Notices") to Aura. You may grant approvals, permission,

extensions, and consents by email. Notices to you may be sent to the email address associated with your account for the Services.

You consent to receive certain electronic communications from us in connection with your use of the Services. You agree that any notices or other communications sent to you electronically will satisfy any legal notice requirements. You must keep contact details associated with your account and accurate, and you will notify Aura in writing of any changes to such details.

2. Contact Us. If you have any questions about these Terms, please contact us at the below address or by contacting us via the phone number or email address in your account dashboard which at the time of publishing is 1-833-552-2123 and support@aura.com:

   Aura Customer Service
   250 Northern Ave.
   3rd Floor
   Boston, MA 02210

2.22 Governing Law/Jurisdiction. These Terms will be governed by and construed by the laws of the Commonwealth of Massachusetts, USA, exclusive of its choice of law principles. OTHER THAN FOR INDIVIDUALS RESIDING IN THE PROVINCE OF QUEBEC, You expressly agree that exclusive jurisdiction for resolving any claim or dispute with Aura or relating in any way to Your use of the Services resides in the state and federal courts of Boston, Massachusetts, and You further agree and expressly consent to the exercise of personal jurisdiction in the state and federal courts of Boston, Massachusetts. You further agree that any such claims will be brought solely on an individual basis and not as part of any class, consolidated, or representative capacity.

Some jurisdictions (such as the province of Quebec, Canada) have laws that require contracts to be governed by the mandatory provisions of the local laws of the consumer's country. In such cases, mandatory provisions of the local laws of your country of residence apply.

2.23 Survival. Upon termination, expiration, or cancellation of the Services for any reason, such terms as by their nature would survive termination shall survive. In particular, Section 2.3 (User Submissions; Feedback; Beta Features; Updates); Section 2.19 (Trademark and Copyright), Section 2.7 (Indemnification), Section 2.9 (Disclaimers; No Warranties); 2.10 (Limitation of Liability and Damages); Section 2.12 (Dispute Resolution by Binding Individual Arbitration), Section 2.22 (Governing Law), Section 2.23 (Survival), Sections 2.24 (Miscellaneous), and Section 4.1 (Ownership) will survive.

2.24 Miscellaneous.

1. Digital Millennium Copyright Act. It is Aura's policy to respond to notices of alleged copyright infringement that comply with the Digital Millennium Copyright Act, and other applicable copyright law. We may in our discretion remove any material alleged to be

infringing and may notify the alleged infringer. For more information, please go to our DMCA Notification Guidelines.

2. Notice to California Residents only. You may reach Aura at the contact information provided below in the “CONTACT US” section. California residents may also reach the Complaint Assistance Unit of the Division of Consumer Services of the California Department of Consumer Affairs by mail at 1625 North Market Blvd., Sacramento, CA 95834, or by telephone at (916) 445-1254 or (800) 952-5210.
3. Modification of this Agreement. Aura may make changes to these Terms at any time and for any reason. You agree that it is your responsibility to regularly check the Services for any updated Terms. The “Last Updated” legend above indicates when these Terms were last changed. If a revision materially alters your rights, as determined by Aura in its sole discretion, we will use reasonable efforts to notify you in advance of the change becoming effective. The most current version of these Terms will be posted on the Services. Changes will be effective no sooner than the last updated date. By continuing to use the Services after the date the changes become effective, you indicate your agreement to be bound by the updated Terms. If you do not agree to any changes made to these Terms, you must immediately stop using the Services.
4. Assignment. These Terms, and any rights and licenses granted hereunder, may not be transferred or assigned by you but may be assigned by Aura without restriction. Any assignment attempted to be made in violation of these Terms shall be void.
5. Severability. If any provision of these Terms is held to be unlawful, void, or for any reason is unenforceable, then that provision will be limited or eliminated from these Terms to the minimum extent necessary and will not affect the validity and enforceability of any remaining provisions.
6. No Waiver. The failure of Aura to exercise or enforce any right or provision of these Terms will not constitute a waiver of such right or provision. Any waiver of any provision of these Terms will be effective only if in writing and signed by a properly authorized representative of Aura. Any cause of action arising out of or related to the Service by you against Aura must commence within one (1) year after the cause of action accrues.
7. Third-Party Beneficiaries. Except as expressly provided herein, there will be no third-party beneficiaries to these Terms.
8. No Relationship. These Terms do not, and shall not be construed to, create any partnership, joint venture, employer-employee, agency, or franchisor-franchisee relationship between you and Aura.
9. Law Enforcement and Public Interest. Nothing contained in these Terms of Use prevent Aura from disclosing your information to third parties if we determine that such disclosure is reasonably necessary to: (a) comply with any applicable law, regulation, legal process, or appropriate government request; (b) protect any person from death or serious bodily injury; (c) prevent fraud or abuse of Aura or our users; or (d) protect Aura’s rights, property, safety, or interest.
10. Interpretation. These Terms were prepared and written in English. Any non-English translations of these Terms which may be made available are provided for convenience only and are not valid or legally binding. Use of section headings in these Terms is for convenience only and will not have any impact on the interpretation of particular provisions. The use of the words "includes," "including," "such as," "for example," and similar terms are deemed not to limit what else might be included.

    (For individuals residing in Quebec only) You acknowledge that a French version of these Terms was remitted to you, and confirm that it is your express wish that these Terms and all related documents and communications be drawn up in English only.

(Pour les individus résidant au Québec seulement) Vous reconnaissez qu'une version française des présentes conditions vous a été remise, et vous confirmez que vous souhaitez expressément que les présentes conditions ainsi que tous les documents et communications connexes soient rédigés en anglais exclusivement.

11. Entire Agreement. These Terms (including any documents incorporated here by reference) constitute the entire agreement between you and Aura concerning its subject matter, and it supersedes any other prior or contemporaneous agreements or terms, written or oral.

Part 3. Service-Specific Terms

Your use of the Services is subject to both the General Legal Terms (see Part 2 above) and these Service Specific Terms. These Service Specific Terms apply to you only if you have purchased or use one or more of the particular Services discussed below. Please note that not all of the Services are available in all countries. If there is a conflict or inconsistency between the General Legal Terms and these Service Specific Terms, the Service Specific Terms will govern and apply. Use of the term “Services” in the remainder of these Service Specific Terms refers to the specific products or services being discussed, as well as any related software offered by Aura.

Your Subscription may include some or all of these Services. It is your responsibility to review these Service Specific Terms and understand which terms and conditions apply to your Subscription. If you have any questions about the applicability of these Service Specific Terms, please feel free to contact us. See Section 2.21 above for Aura’s contact information.

Any warranty disclaimers or limitations of liability included in these Service Specific Terms do not limit the applicability or force of any more general warranty disclaimers or liability limitations found elsewhere in these Terms, including, without limitation, Sections 2.9 and 2.10 of the General Legal Terms.

3.1 Dark Web Monitoring. With our Dark Web Monitoring feature (“Dark Web Monitoring Feature”) as part of the Services, you can designate your financial accounts, mailing address, and other types of personal information, up to the limits set forth in the Services. This Dark Web Monitoring Feature monitors for the information that you designate through your account (and nothing else) (“Dark Web Monitoring Designated Information”) and notifies you when your Dark Web Monitoring Designated Information is found online. You acknowledge and agree we cannot remove your Dark Web Monitoring Designated Information from the dark web and that we do not ensure the accuracy or integrity of the information on the dark web. You also acknowledge and agree that the Dark Web Monitoring Feature will not identify all instances of your Dark Web Monitoring Designated Information on the dark web and any alleged failure to identify Dark Web Monitoring Designated Information on the dark web will not constitute a breach or default by us of our obligations.

By entering the Dark Web Monitoring Designated Information, you confirm that you own this information and have the right to provide it to us and agree not to use the feature for any purpose other than identity theft protection

3.2 Secure VPN. Our Secure VPN Services generally provide sufficient capacity to accommodate average non-commercial use. It is possible that you may temporarily experience slower service or service unavailability. The accuracy and timeliness of data received is not guaranteed; delays or omissions may occur. Aura is not responsible for data, messages, or pages lost, not delivered, delayed, or misdirected because of interruptions or performance issues with the Secure VPN Services, communications services, or networks. Any such slowdown, unavailability, or loss of data will not constitute a breach or default by us of our obligations.

We may impose usage or service limits, suspend services, or block certain kinds of usage at our sole discretion to protect Aura, our Customers, or the Services. We will not be liable to you or owe you any refund or other compensation as a result of any such actions. We do not condone or endorse any unlawful, illicit, criminal or fraudulent activities perpetrated by you while using the Services. We will not be liable in any way for any actions of the users of the Services. Aura reserves the right to prevent your access to the Service or continued use thereof if you violate this Agreement, engage in fraud or copyright infringement.

3.3 Identity Theft Protection Services. The Identity Theft Protection Services, including features such as monitoring your credit profile, providing insurance for eligible identity theft claims, and monitoring certain publicly available registries maintained by local law enforcement agencies for your information, are only available to users who have a U.S. Social Security Number and a U.S. mailing address. You agree that you will use these Services only for your own behalf, except if you are enrolled in the Family Plan in which case each adult member of the Family Plan agrees to the requirements in this Section. You will be responsible for all use of your membership number and if applicable for a Junior Member that you enroll for Identity Theft Protection services under the Family Plan, for any Junior Member's membership number (together, "Membership Number"). You must notify Aura immediately of any unauthorized use of any Membership Number, or the theft or misplacement of a Membership Number.

Authorization: As needed to provide the Services to You, You authorize and instruct Us to obtain, monitor and compile your (a) credit information from one or more credit reporting agency(-ies), and (b) "non-public personal information", "personal information," and "highly restricted personal information" about you as defined by the Gramm-Leach-Bliley Act (15 U.S.C. §6801 et seq.) and Driver's Privacy Protection Act (18 U.S.C. § 2721 et seq.), respectively, and other personal information. You understand that by enrolling in the Services, you are providing "written instructions" in accordance with the applicable law, including the US federal Fair Credit Reporting Act, as amended ("FCRA"), for Aura and its service providers, which may include CSIdentity Corporation ("CSID"), Equifax Inc. ("Equifax"), Experian, Inc. ("Experian"), and Transunion, LLC ("TransUnion") to obtain information from your personal credit profile from Experian,

Equifax, and Transunion, the three major credit reporting agencies. If applicable, you authorize Aura and its service providers to use your Social Security number to access your personal credit profile, verify your identity, and provide credit monitoring, reporting, and scoring products. Further, you authorize Aura to make any inquiries necessary to verify your identity or your ownership of financial or other accounts relevant to your use of the Services. This may include, without limitation, making inquiries through third-party sources or requiring you to provide additional information or documentation. If we are unable to verify your identity or otherwise obtain your credit information from a credit reporting agency, we will be unable to provide certain Services to you and reserve the right to refuse to offer some or all of the Services to you, downgrade or cancel your Subscription, as applicable.

Insurance: Identity theft insurance is governed by a master policy (which may differ depending on the identity theft insurance plan you are enrolled in), the terms of which are incorporated herein by reference. The issuance of the policy is evidenced by the applicable insurance certificate, summary of benefits, or other similar document that has been provided to you. You acknowledge receipt of this information and agree to be bound by the terms, conditions, limitations, and exclusions of the policy applicable to the identity theft insurance plan you are enrolled in.

Credit Report-Related Disclosures for US Residents: The Fair Credit Reporting Act allows you to obtain a disclosure from every credit reporting agency of the nature and substance of all information in your file at the time of the request. Full disclosure of information in your file at Experian must be obtained directly from Experian by calling 888-397-3742 or logging on to www.experian.com/consumer. The credit report you are requesting from Aura is not intended to constitute the disclosure of Experian information required by the Fair Credit Reporting Act or similar state laws. You are entitled to receive a disclosure directly from the consumer reporting agency free of charge under the following circumstances:

- You have been denied credit, insurance or employment within the past sixty (60) days as a result of your credit report.
- You certify in writing that you are unemployed and intend to apply for employment in the 60-day period beginning on the date on which youmade the certification.
- You are a recipient of public welfare assistance
- You have reason to believe that your file at the agency contains inaccurate information due to fraud Annually at www.annualcreditreport.com

Otherwise, subject to applicable law, the consumer reporting agency may impose a reasonable charge for the disclosure.

The Fair Credit Reporting Act permits you to dispute inaccurate or incomplete information in your credit file. You understand that accurate information cannot be changed. You do not have to purchase your credit report or other information from Aura to dispute inaccurate or incomplete information in my Experian file or to receive a copy of your Experian consumer credit report.

Experian’s National Consumer Assistance Center provides a proprietary consumer disclosure that is different from the consumer credit report provided by Aura. The disclosure report must be obtained directly from Experian. Consumers residing in the States of Colorado, Massachusetts, Maryland, New Jersey, and Vermont may receive a free copy of their consumer credit report once per year and residents of the State of Georgia may receive two copies per year.

Fraud Alerts are available to any eligible consumers—free of charge—from a national consumer reporting agency.

NOTICE TO ILLINOIS RESIDENTS: MANY GOVERNMENT RECORDS ARE AVAILABLE FREE OR AT A NOMINAL COST FROM GOVERNMENT AGENCIES. CREDIT REPORTING AGENCIES ARE REQUIRED BY LAW TO GIVE YOU A COPY OF YOUR CREDIT RECORD UPON REQUEST AT NO CHARGE OR FOR A NOMINAL FEE.

The Services are not intended to substitute for any free credit report or disclosure that any credit reporting agency or bureau is required by law to provide to you. Neither you nor anyone else has the right to have accurate and current information removed from your credit report. If information in your credit report is inaccurate, you have the right to dispute it by contacting the credit bureau directly.

VANTAGESCORE 3.0 CREDIT SCORE: VantageScore 3.0, with scores ranging from 300 to 850, is a user-friendly credit score model developed by the three major nationwide credit reporting agencies, Experian®, TransUnion®, and Equifax®. VantageScore 3.0 is used by some but not all lenders. Higher scores represent a greater likelihood that you'll pay back your debts so you are viewed as being a lower credit risk to lenders. A lower score indicates to lenders that you may be a higher credit risk.

There are three different major credit reporting agencies, Experian, TransUnion, and Equifax, that maintain a record of your credit history known as your credit file. Credit scores are based on the information in your credit file at the time it is requested. Your credit file information can vary from agency to agency because some lenders report your credit history to only one or two of the agencies. So your credit scores can vary if the information they have on file for you is different. Since the information in your file can change over time, your credit scores also may be different from day-to-day. Different credit scoring models can also give a different assessment of the credit risk (risk of default) for the same consumer and same credit file.

There are different credit scoring models which may be used by lenders and insurers. Your lender may not use VantageScore 3.0, so don't be surprised if your lender gives you a score that's different from your VantageScore. (And your VantageScore 3.0 may differ from your score under other types of VantageScores). Just remember that your associated risk level is often the same even if the number is not. For some consumers, however, the risk assessment of VantageScore 3.0 could vary, sometimes substantially,

from a lender's score. If the lender's score is lower than your VantageScore 3.0, it is possible that this difference can lead to higher interest rates and sometimes credit denial.

Certain Public Records Monitoring: The sex offender ("Offender") information that is used for a specific feature for the Services to monitor for if your information is added to a sex offender registry is derived from official public records. As such, the accuracy of the information provided through the Services may vary based upon the accuracy of such official public records. In certain cases, we may not obtain all the address information associated with the Offender who is contained in the public record. Aura has no control over the contents of official public records. If you believe the information found using this feature is incorrect, please contact the local police department in the applicable jurisdiction for assistance. That police department will be able to assist you directly or refer you to the appropriate authority.

3.4 Data Broker Opt Out. If you sign up for the Data Broker Opt Out Services, you acknowledge and agree to the following:

1. On your behalf, Aura will make reasonable efforts to have your personal information that you provided to us removed or suppressed ("opt-out"), in whole or in part, certain third-party databases and/or websites (the "Data Brokers") identified in the Services. You understand that we will only be able to submit opt outs for the information you provide to us.
2. You expressly authorize Aura, its agents, and its employees, to act as your personal representative or authorized agent to submit opt outs to Data Brokers and otherwise implement the Services and if applicable and available to you, for any Junior Members under the Family Plan, including by acting to:

- obtain information on your behalf;
- submit your personal information to Data Brokers;
- communicate with Data Brokers or other third parties on your behalf;
- take any other actions that Aura believes is reasonably necessary to opt you out of a Data Broker.

3. The extent of the opt-out will vary based on the Data Broker, and its terms and practices. It is possible that some Data Brokers may contact you to confirm your request and/or to request additional information; and in such an event, our ability to remove your information will depend on your response to them.
4. You can personally opt out to these Data Brokers to the same extent that Aura submits the opt out on your behalf. Aura makes no claims that it has any ability to opt you out of any Data Broker beyond your ability to do so on your own behalf.
5. Aura is under no obligation to and will not pay any fees to a Data Broker in order to opt you out.
6. You must give us authority to act only on your behalf and not on behalf of any person other than yourself except if applicable and offered to you, as a feature under the Family Plan for a Junior Member. If you give us information that we know, discover, or suspect is false or inaccurate, or relates to another person that is not accurate, we may suspend or terminate your access to the Services and reserve the right to, where we deem it

appropriate, report such conduct to law enforcement and other third parties, and to cooperate in the investigation of such conduct.

7. We are not liable to you for the manner in which a Data Broker treats or uses your personal information. A Data Broker's treatment or use of your information will be subject to the Data Broker's policies, if any, and not these Terms or the Aura Privacy Policy.
8. While we will make reasonable efforts to remove your information, the success of the Services is ultimately dependent on the cooperation of the Data Brokers. We cannot and do not guarantee that the Services will be effective, that opt out requests will be honored by Data Brokers, or that all opt outs will be equivalent (e.g., some Data Brokers may remove all of the information included in the opt out submitted, while other may remove only some or none at all). We are not responsible for the actions or inactions of any Data Broker, and make no representations, warranties or guarantees related to such. You expressly agree that we will not be liable to you under any circumstances for any actions or inactions of the Data Brokers, or for our failure, for any reason, to effectuate opt outs on your behalf.
9. You may choose to install the Aura browser extension on a supported browser to run the Broker Opt Out Services on your computer. By installing and activating the Aura browser extension, you expressly authorize Aura, its agents, and its employees, to submit opt out requests to Data Brokers for you from your device. You may revoke this authorization at any time within the Aura browser extension.

3.5 Social Media Monitoring. Our Social Media Monitoring Services will monitor your accounts on certain social media sites and notify you of any suspicious activity or potentially malicious links. We will only monitor accounts that you connect to the Services.

3.6 Financial Transaction Monitoring. If you choose to activate our Financial Transaction Monitoring Services, you must provide us with any necessary information to render the Services, including, without limitation, the applicable account credentials for your credit, commerce, banking, investment, and other eligible account(s) ("Transaction Account Credentials"). Should you change your account information (including your Transaction Account Credentials with any of your third-party providers) you must also update that account information in our system in order for Aura to continue to provide the Services to that respective account. You hereby acknowledge and understand that our network(s), products, and/or services are limited to the account information provided by you and your third-party providers, and we may in certain limited circumstances experience delays and/or failures to process or identify fraudulent transactions. You hereby acknowledge and agree to monitor your accounts and transactions and further expressly recognize that you should not solely rely on alerts from Aura for accuracy or delivery in all cases.

3.7 Additional Title Monitoring. We may offer additional title monitoring for property you own and register with us such as for your home or car ("Additional Title Monitoring"). Our Additional Title Monitoring services is a notice-only service that sends a notification to you if we identify an ownership or other change on the title to your monitored property based on the applicable records kept by the relevant authorities such as by your county's assessor or recorder's office ("Authorities"). If you receive a notification, you will need to contact your relevant authority to get more detailed

information and/or correct their records. The accuracy of the notifications provided through the Services is dependent on the Authorities' records. You acknowledge and agree that we will not be liable for any inaccuracies or errors in such records, or for the absence of any information in such records.

3.8 Identity Theft Remediation. In order for us to provide our Identity Theft Remediation Services, you must provide us with a signed Limited Power of Attorney ("LPOA") in the form that we will send to you. A LPOA form may be sent to you after you contact our customer support team which you will be required to complete and return in accordance with the instructions enclosed. The LPOA allows us to work on your behalf with creditors, merchants, banks and other entities. You may also be required to provide us with other documents relating to the applicable identity theft event, depending on the circumstances.

In addition, separate and apart from the LPOA, you authorize us to take all reasonable actions on your behalf to help address your identity theft incident. You acknowledge and agree that you do not have to specifically authorize each action we take, and you further acknowledge that we can investigate the facts and circumstances related to your identity theft case, including but not limited to contacting third parties by mail, telephone, and email. We reserve the right to ask for evidence of or related to the identity theft event, including but not limited to: affidavits, police reports, and/or other government reports.

3.9 Additional Members; Alert Sharing. Some Services, such as our Family Plan, may allow you to register your parents or legal guardians, parent in-laws, sibling in-laws, conservatees, spouses/domestic partners, or minor children (for whom you are the legal guardian or parent ("Family Member(s)") or their devices to use the Services, enable you to add other adults, enter in information about the family member, or upload documents on their behalf, or help you monitor and manage the online activities of children up to 18 years old. Applicable Services may be only accessed and used by, or on behalf of, Family Members . If you are enrolling a Family Member or using the Services on their behalf, you agree that the information you provide to Aura about yourself or about the third-party is true and accurate. You acknowledge and agree that You will not enroll any Family Member and/or monitor their accounts unless you obtain express consent from and are duly authorized by the Family Member and such consent and authorization is documented by You. By enrolling a Family Member in the Services, You acknowledge that you are subject to these Terms and responsible for the Family Members' activity on the Services.

If requested, you agree to provide documentation necessary in our sole discretion to prove your relationship with any minor child or other people on your account and any authorizations and/or consents obtained. You further acknowledge and agree that subject to our then-current customer authentication procedures, another adult customer enrolled on your account may authorize changes to the account, including termination of your subscription or changes resulting in additional charges.

No single member of a Family plan can view or update the accounts for his/her entire household through his or her own account. Each account holder (or a parent/legal guardian for minors) must sign into his/her own account in order to view or update their information. If you have additional members in your subscription plan, you may be able to elect to share certain information or alerts you received from the Services with members of your choosing (“Alert Sharing”). You may choose to end future Alert Sharing with any member in your plan at any time. Information shared prior to revoking Alert Sharing may still be accessible by the member in your Family Plan. If you receive information or alerts related to an additional member’s credit profile (“Credit Information”), you agree you will not use such Credit Information for consumer or commercial credit-granting purposes, and in all cases will use such information in accordance with all applicable laws and regulations. Aura is not responsible for the actions of individuals whom you choose to share your informational alerts with.

3.10 Parental Controls. The parental controls Services (“Parental Controls”) are only for parents to monitor the Internet and mobile activity of their minor children and are designed to be administered by adults on behalf of a family or community. If you are subscribing to the Parental Controls, you are giving the undertakings and consents contained in these Terms and the Privacy Policy **on behalf of your own child(ren) and legal dependent(s)**. Information collected by Aura to provide the Parental Controls to you is more fully described in the Aura Parental Controls Privacy Notice. **BY USING THE PARENTAL CONTROLS SOFTWARE, YOU EXPRESSLY AUTHORIZE AURA TO PROCESS THIS INFORMATION ON YOUR BEHALF, AND INDEMNIFY AURA FROM ANY THIRD PARTY CLAIMS, INCLUDING ANY STATE OR FEDERAL ‘WIRETAP’ LAW CLAIMS, ASSOCIATED WITH THE COLLECTION OF THIS INFORMATION ON YOUR BEHALF.**

You may use the Services to track and monitor only your minor children, minor children for whom you are the legal guardian or others for whom you have legal authorization to track and monitor. By enrolling a child as part of the Family Plan, you represent and affirm that (a) you are the custodial parent or legal guardian of each minor child you register, (b) any documentation that you provide asserting the same is valid, (c) each minor child that you register legally resides at your address of record, and (d) that as required, you are able and willing to provide a copy of (i) your valid driver's license, (ii) your child's social security card, (iii) your child's birth certificate, and (iv) any other documentation required to verify proof of legal guardianship. You agree to notify us in the event that the status of your legal guardianship changes; we reserve the right to cancel each child upon such status change. Enrollment of any child in Family Plan will be canceled upon any child becoming 18 years old and the primary member may then invite the adult (former child) to join his/her Family Plan. If requested, you agree to provide documentation necessary in our sole discretion to prove your relationship with any minor child on your account.

The parent or legal guardian user(s) in a Family Plan with Parental Controls selected are solely responsible for any activity or actions in their Family Plan account resulting from the use of their log-in credentials on the Services, whether or not the primary user

has authorized such activities or actions. **You acknowledge that the Parental Controls feature does not monitor for all content or your child's behavior in real time. Alerts and/or insights may not be 100% accurate or timely. Any information obtained through your use of the Parental Controls is delivered to you for your use at your own discretion and risk. No advice, results, or information or materials, whether oral or written, or obtained by you through the Parental Controls shall create any warranty or representation by Aura.**

You further acknowledge that Aura does not guarantee that content you deem objectionable will be 100% unavailable at all times or at any time. You assume full risk and responsibility for the use of or reliance on the Parental Controls feature as regards content blocking. "False positive" content blocking may occur from time to time or at any time. We strive to allow sufficiently granular control of content filters to allow educational or meritorious content through, if that is the intent of the user. However, there is no guarantee that some content that you would deem acceptable will not be blocked by the Parental Control feature. In the event that you believe we are miscategorizing a site or service, please contact us at support@aura.com to submit the issue for review.

PRECAUTIONS:

THE PARENTAL CONTROL SERVICES ARE NOT A MEDICAL DEVICE AND YOU EXPRESSLY AGREE THAT THE SERVICES DO NOT INVOLVE THE PROVISION OF MEDICAL ADVICE BY AURA. THE SERVICES ARE NOT INTENDED TO DIAGNOSE, TREAT, CURE, OR PREVENT ANY DISEASE OR MEDICAL CONDITION. THE SERVICES ARE FOR INFORMATIONAL PURPOSES ONLY AND CANNOT REPLACE THE SERVICES OF PHYSICIANS OR MEDICAL PROFESSIONALS.

THE PARENTAL CONTROL SERVICES, INCLUDING ALL INFORMATION, TEXT, PHOTOGRAPHS, IMAGES, ILLUSTRATIONS, GRAPHICS, AUDIO, VIDEO, AND AUDIO-VIDEO CLIPS, AND OTHER MATERIALS, WHETHER PROVIDED BY US OR THIRD PARTIES, IS NOT INTENDED TO BE AND SHOULD NOT BE USED IN PLACE OF (A) THE ADVICE OF YOUR PHYSICIAN OR OTHER MEDICAL PROFESSIONALS, or (B) A VISIT, CALL, OR CONSULTATION WITH YOUR PHYSICIAN OR OTHER MEDICAL PROFESSIONALS.

SHOULD YOU HAVE ANY HEALTH-RELATED QUESTIONS REGARDING YOUR CHILD, PLEASE CALL OR SEE YOUR CHILD'S PHYSICIAN OR OTHER MEDICAL PROVIDER PROMPTLY. SHOULD YOU HAVE AN EMERGENCY, CALL YOUR PHYSICIAN OR 911 IMMEDIATELY. YOU SHOULD NEVER DISREGARD MEDICAL ADVICE OR DELAY IN SEEKING MEDICAL ADVICE BECAUSE OF ANY INFORMATION PRESENTED ON THE SERVICES, AND YOU SHOULD NOT USE THE SERVICES OR ANY INFORMATION PROVIDED IN THE SERVICES FOR DIAGNOSING OR TREATING A HEALTH PROBLEM. THE TRANSMISSION AND RECEIPT OF SERVICES, IN WHOLE OR IN PART, OR COMMUNICATION VIA THE INTERNET, EMAIL, OR OTHER MEANS DOES NOT CONSTITUTE OR CREATE A

DOCTOR-PATIENT, THERAPIST-PATIENT, OR OTHER HEALTHCARE PROFESSIONAL RELATIONSHIP BETWEEN YOU AND AURA.

3.11 Anti-Virus/Malware Removal. If your Subscription includes our Anti-Virus or Malware Removal Services, or a similar service whereby we or one of our partners access your device to attempt removal of malware or perform other specified services, additional terms may apply. Such terms may be specified in the documents that accompanied your purchase.

We will make commercially reasonable efforts to perform the malware removal Services covered by your Subscription. You understand and agree that not all malware can be removed through the Services and we do not guarantee that we can remove all malware from your device(s). To the extent permitted by applicable law, we will have no liability for loss of or recovery of data, software, or loss of use of system(s) or networks arising out of your use of these Services or any act or omission, including negligence, by us or our representatives. You agree that you have a valid and legal license to access and download the Services on your device(s) and that your use of the Internet is solely at your own risk. By electing to receive the Services, you confirm that you (i) have full access to your hardware and software for which you are purchasing the Services, and (ii) have completed a back-up onto separate media of any software or data on the hardware that may be impacted by the Services.

3.12 Safe Browsing. Our Safe Browsing Services provide information to guide users about certain risks that may be associated with a website. We do not control or assume responsibility for the content of the third-party sites, and some of the third-party sites may have content that you find objectionable, inappropriate, or offensive. THE SITE RATINGS PROVIDED THROUGH THE SERVICES ARE NOT A GUARANTEE OF ANY PARTICULAR SITE’S SPECIFIC PRACTICES OR TRUSTWORTHINESS, AND IN NO CASE DO THE RATINGS REPRESENT AN ENDORSEMENT BY US OF THE SITE’S CONTENT, SUBJECT MATTER, OVERALL QUALITY, OR USEFULNESS.

3.13 Mobile Messaging Alerts. Our Mobile Alert Services allow you to receive informational alerts related to the categories you select. By providing your mobile telephone number to Aura when presented with or informed of these Terms, you consent to receive non-marketing mobile messages to the fullest extent permitted under applicable law, regardless of the technology utilized. These messages are provided when activity on one of your accounts is detected and triggers a requested alert, as well as to provide information on billing matters. The type and frequency of alerts you receive through SMS may differ from the alerts you receive within the App, and mobile alerts may be delayed, incomplete, or not received due to technical errors from our systems, the mobile provider, a third-party company, or your mobile service or device. We expressly disclaim any and all liability related to failure to, accurately, fully, or timely deliver any mobile alerts. You can cancel the Mobile Alert Services at any time. Just text "STOP" to our short code. After you send the SMS message "STOP" to us, we will send you an SMS message to confirm that you have been unsubscribed. The Services may recognize or respond to additional commands and keyword queries. Thus, you may

receive additional informational text messages based on your interaction with the Services, even after opting out of receiving the mobile alerts. You acknowledge and agree that, notwithstanding any prior opt-out attempt, you consent to receive further messages from or on behalf of Aura that result from your continued communication with the Services. Aura may also provide you instructions on how to rejoin receiving the Services' recurring mobile alerts when you unsubscribe. If you are experiencing issues with the messaging program you can reply with the keyword HELP for more assistance, or you can get help directly at 1-833-552-2123. Carriers are not liable for delayed or undelivered messages. As always, message and data rates may apply for any messages sent to you from us and to us from you. Message frequency will vary. If you have any questions about your text plan or data plan, it is best to contact your wireless provider. If you have any questions regarding privacy, please read our privacy policy.

3.14 Alias Email. Our Alias Email Services allow you to generate a random, "masked" email address to use when creating account credentials for third-party sites and services online. Emails sent from the third-party service provider to the masked email address will be redirected to the email address associated with your Subscription. As such, the Services will not prevent your receipt of any unwanted emails sent by third parties. The sole function of the Services is to permit you to help keep your email address private when creating an account online with a third-party. We do not guarantee that the Services will be effective or functional for all third-party sites and services.

3.15 Experian CreditLock. By requesting an Experian CreditLock to be placed on your behalf, you are requesting to restrict most third-party access to your Experian credit report. You acknowledge that your Experian credit report, when locked with Experian CreditLock, may still be accessible by third parties in certain instances deemed to be low-risk by Experian, including access to your Experian credit report by the following third parties: (1) You, as part of any Service offering Experian CreditLock as a benefit; (2) potential employers or insurance companies; (3) companies that have an existing credit relationship with you; (4) collection agencies acting on behalf of companies or individuals on a debt you may owe and related collection activities; (5) companies providing pre-screened credit card offers. Please understand that your Experian credit report will be unlocked if you cancel a Service that includes Experian CreditLock as a benefit, or downgrade to a Service that does not include Experian CreditLock as a benefit.

Your Experian CreditLock will restrict delivery of your Experian credit report only, and does not apply to your TransUnion or Equifax credit report. To lock or place a security freeze on your Equifax or TransUnion credit report, you will need to contact those bureaus directly. While Experian CreditLock may include some functionality similar to security freeze programs required under certain laws, you acknowledge that Experian CreditLock is a separate service from, and not equivalent to, such security freeze programs. For more information about the Experian security freeze program, please contact Experian at https://www.experian.com/freeze/center.html or 1-888-EXPERIAN.

3.16 Vault. If your subscription includes Aura's Vault feature, you may use it to store your documents, certain personal information of yours, your passwords for any websites, apps or services that you elect to use it for, or other information ("User Information") and scan the dark web via the Dark Web Monitoring Feature. . The amount of storage space Aura provides to you is determined by the plan you select. If you exceed your allotted amount of storage space, we will restrict you from uploading additional User Information. Such restrictions shall remain until any such excess usage is adequately eliminated by you. By using the Services, you confirm and agree that Aura's use of your User Information as described in these Terms does not and shall not require any payment to any person or entity and does not require license, authorization, approval or consent by any other person or entity.

We may enable you to elect to share this User Information with other individuals in your family plan or third-parties who do not have an account with Aura via a secure web link or, if the individual has an account with Aura, within the Aura Vault ("Vault Sharing"). Sharing within the Aura's Vault may be revoked by you at any time, however, if you choose to send User Information via a secure web link and the recipient downloads the Shared Information to their device, Aura cannot guarantee the individual receiving the Shared Information no longer has access to it. By electing to share your User Information, you acknowledge that the individual receiving the User Information may share the User Information with other individuals either through the Services or outside of the Services. Aura is not responsible for the actions of individuals whom you choose to share your documents, personal information, and passwords with.

Our Aura Vault browser extension will periodically notify you to store your passwords with Aura when you're browsing the Internet. We recommend that you do not share the password to your Aura account with anyone, because anyone with access to your Aura account may be able to obtain access to your other passwords that you do not wish to share.

3.17 Call Protection and SMS Spam Blocker. If your Aura subscription includes Aura's Call Protection and SMS Spam Blocker, the feature will enable you, among other things, to automatically block nuisance calls (robocalls, telemarketers, etc.), to forward all unknown calls to an AI assistant ("Call Assistant") to blacklist and whitelist specific numbers and, if enabled, to block SMS spam messages. Aura also offers automated call screening assistance. When an unrecognized number calls that is not (i) a contact, (ii) on your whitelist, or (iii) not clearly identified as spam, the automated call screening assistance feature will prompt the caller to state a name and purpose for the call, all of which is transcribed and made available for your review. Certain features may not be available in earlier versions of the application or on Android devices.

Functionality: We use a telephone carrier network ("PSTN") to deliver calls to your Device. The technology and how calls are received on your Device are determined by your telephone carrier and the version of the operating system your Device is running when the App is set up. Please note that you are solely responsible for any charges

imposed by your mobile service provider for (i) using the call forwarding feature, (ii) local or long distance usage, and (iii) text message and data usage.

Call Blocking/Forwarding: Activating the call blocking/forwarding feature means that certain incoming calls (e.g. declined calls, silenced calls or unanswered calls) are forwarded to Aura in order to identify and block nuisance and unwanted calls accordingly (or send them back to your Device as a safe call - all before your phone rings). Calls from your Device's contacts go directly to your Device.

Number Monitoring: You agree to check the numbers and messages from blocked calls on your Device regularly to ensure that you whitelist any legitimate phone number so that it will not be blocked in the future. The Service also provides you the ability to create and modify a personal whitelist ("Allow List") and blacklist ("Block List"). You can use the whitelist feature to ensure that a specific number is never blocked. You can add or remove users from your personal Allow List and Block List at any time. Please note that if you have requested a call from, or have an existing relationship with a debt relief company, collections company or if you provided your number to authorize a sales call to any company and you desire to continue receiving those calls, we advise you to add the number to your contacts or Allow List and to monitor your Aura messages for calls you may wish to return.

Access to Contacts: In order to provide the Services, Aura must access the numbers in your Device's address book.

Call Assistant. Aura provides you the option to use its Call Assistant to respond to all unknown calls. If you choose to use Call Assistant, unknown callers (i.e. those not in your phone contact list) will hear a very polite but concise default recording. When the unknown caller engages with Aura's Call Assistant, the conversation is recorded and transcribed. You may also choose to have the Call Assistant listen to and monitor your conversations with unknown callers ("Active Call Assistant Monitoring Feature"). If you activate the Active Call Assistant Monitoring Feature, the Call Assistant will alert you of potential scams if the Call Assistant determines the unknown caller's conversation with you is suspicious. Please note that if you activate the Active Call Assistant Monitoring Feature your conversation with any unknown caller will be recorded and transcribed.

The Call Assistant will categorize the call as Verified, Spam, or Unverified. "Verified" means the Call Assistant determines the unknown caller is not making an unsolicited commercial call (i.e. a doctor's appointment reminder). "Spam" means the Call Assistant determines the unknown caller is making an unsolicited commercial call or a known scam offer (e.g. a call from a suspected scammer to extend your car's warranty). "Unverified" means the unknown caller's response is too vague for the Call Assistant to categorize the call as Verified or Spam.

By default, if the Call Assistant categorizes the call as Verified, it will transfer the call to you. If the Call Assistant categorizes the call as Spam or Unverified, it will request the caller leave a voicemail, which will be recorded and saved within the App ("Call

Assistant Recording"). You choose to change these default settings to have the Call Assistant also transfer Spam and/or Unverified calls to you. BY USING AURA'S CALL ASSISTANT FEATURE, YOU EXPRESSLY AUTHORIZE US TO RECORD AND TRANSCRIBE CALL ASSISTANT CONVERSATIONS ON YOUR BEHALF.

If made available by Aura, you may choose to have the Call Assistant listen to and monitor your conversations with unknown callers ("Active Call Assistant Monitoring Feature"). If you choose to activate the Active Call Assistant Monitoring Feature, the Call Assistant will alert you of potential scams if the Call Assistant determines the unknown caller's conversation with you is suspicious. Please note that if you activate the Active Call Assistant Monitoring Feature your conversation with any unknown caller will be recorded and transcribed.

Legal Compliance: Depending on the state or country in which you are located, the Call Assistant recordings may require you or Aura to acquire the consent of the other parties on the call prior to recording the telephone conversation. YOU ACKNOWLEDGE AND AGREE THAT AS THE INDIVIDUAL DIRECTING THE RECORDING, YOU ARE SOLELY RESPONSIBLE FOR DETERMINING THE LAW IN THE STATE OR COUNTRY IN WHICH YOU ARE RECEIVING CALLS PRIOR TO USING THE CALL ASSISTANT FEATURE IN THAT STATE OR COUNTRY. You agree not to enable the Call Assistant feature unless you have first determined that your use is legal.

Voicemail and Call Screening Transcriptions: Aura may transcribe voicemail messages and call screening recordings that will be made available for your use. Should you decide to deploy the transcription features, you acknowledge and expressly agree that Aura may transcribe or have transcribed by a third party and store or have stored by a third party, voicemail messages and call screening recording, and to make such transcriptions available to you.

Call Data Retention: We make no guarantees that we will retain certain call data such as calls, call recordings, voicemail, call screens, Call Assistants and transcriptions for greater than six (6) months, after which we may delete such call data. If you would like to retain this information, you can go to the App's call details and share the information to your email or cloud storage account. You acknowledge that you bear sole responsibility for the back-up of this information.

SMS Spam Protection: Aura provides you with the option to enable a SMS spam protection feature which will filter SMS and MMS messages from individuals not in your Device's contacts. When you enable the SMS spam blocking feature and grant us permission, we will filter SMS text messages from individuals that are not in your Device's contacts and attempt to block any spam messages.

Call Forwarding Deactivation: Please follow the following deactivation steps if you enabled the call blocking/forwarding feature during the setup process. In addition to unsubscribing, you must deactivate your account within the App. If you still have the App on your Device, please open the Aura App > tap the *settings* tab located in the

bottom right > tap *help & feedback* under *support* > tap *disable Aura* > follow the on-screen instructions. If you deleted the App before deactivating the call blocking/forwarding feature, please open the *settings* icon on your Device > tap *phone* > tap *call forwarding* > turn off call blocking/forwarding. If you fail to deactivate the call blocking/forwarding feature, you will not receive your voicemail messages and may experience other problems with your telephone carrier.

3.18 Email Protection. If your Aura subscription includes Aura's Email Protection, you will be able to connect your third-party email accounts to Aura. Once connected, Aura will access, monitor, review, and store each email you receive to assess and notify you whether the email may be a scam or not. You acknowledge that use of the Email Protection feature does not guarantee that all scam e-mails will be identified 100% of the time. You assume full risk and responsibility for the use of or reliance on the Email Protection results as it relates to identifying scams. "False positives" and may occur from time to time or at any time.

Part 4. Limited License

We are pleased to grant you, an individual user, a personal, non-transferable, nonexclusive term-limited and revocable license to install and use the Software and access the Services for which you have purchased a Subscription for personal use on the number of Devices (defined below) and for the number of users specified in your License Entitlement or Service Entitlement, as applicable, subject to the System Requirements.

"License Entitlement" means the number and type of Devices and users that are permitted to download and use the Software and access the Services, as specified at time of purchase and in your account profile. If no licensed device count or user count was specified, the License Entitlement is for a single Device and user.

"Service Entitlement" means the scope and duration of the Services you purchased, as specified at time of purchase and in your account profile. If no scope or duration is specified in the documents, the Service Entitlement is for a single Device and user for one year.

"System Requirements" means the supported Devices and operating systems that the particular Aura product or service you purchased will function properly with, as listed on our website or other applicable documentation. It is your responsibility to meet System Requirements, such as obtaining updates or upgrades to continue using the Services.

4.1 Ownership; Proprietary Rights. The Services, including the Software (and any releases, revisions, updates, or enhancements), and all of their content, features, and functionality, including, without limitation, information, text, graphics, logos, button icons, images, visual interfaces, audio clips, video clips, data compilations, computer code (including source code or object code), software, products, services, and the design, selection and arrangement thereof, and any accompanying documentation (collectively,

the “Materials”) are the exclusive property of Aura, its licensors, or other content suppliers and are protected by the United States and Canadian copyright, trade dress, patent, and trademark laws, international conventions, and other relevant intellectual property and proprietary rights laws. Except as expressly authorized by Aura, you agree not to sell, license, distribute, copy, modify, publicly perform or display, transmit, publish, edit, adapt, create derivative works from, or otherwise make unauthorized use of any of the Services or Materials. Aura reserves all rights not expressly granted in these Terms.

4.2 Limited License. Subject to these Terms, Aura grants you a limited, revocable, nonexclusive, personal, nontransferable license to install and use the Software and to access the Services for internal, noncommercial, and personal purposes only and subject to your Service Entitlement and License Entitlement. The Software is “in use” on a Device for purposes of this paragraph when it is loaded into the temporary memory (e.g., RAM) or installed into the permanent memory (e.g., hard disk, CD-ROM, or another storage device) of the Device.

4.3 License Restrictions. You may not (and may not allow a third-party to):

1. reverse engineer, decompile, disassemble or attempt to reconstruct, identify or discover any source code, underlying ideas, underlying user interface techniques or algorithms of the Services by any means whatsoever, except to the extent that such restriction is expressly prohibited by applicable law;
2. remove or destroy any copyright notices or other proprietary markings from the Services;
3. attempt to circumvent any use restrictions applicable to the Services;
4. modify or adapt any aspect of the Services, merge any aspect of the Services into another program, or create derivative works based on the Services;
5. use, copy, or distribute the Software without Aura’s written authorization, except that you may make one (1) copy of the Software for archival or backup purposes only.
6. use the Services to provide, alone or in combination with any other software, product or service, any software/product/service to any person or entity, whether on a fee basis or otherwise or on websites where you have agreed "not to use any ad blocking solutions", for instance by agreeing to the terms of services on the respective websites or to circumvent technological measures that control access to websites;
7. repackage, modify, adapt, tamper with, alter, translate, or create derivative works of the Services or any aspect thereof;
8. combine or merge any part of the Services with or into any other software or documentation, or refer to or otherwise use the Services as part of an effort to develop software (including, without limitation, any routine, script, code, or program) having any functional attributes, visual expressions, or other features similar to those of the Services or to compete with Aura;
9. except with Aura’s prior written permission, publish any performance or benchmark tests or analysis relating to the Services;
10. rent, lease, sublicense, sell, assign, loan, use for timesharing or service bureau purposes, or otherwise transfer the Services or any of your rights and obligations under this Agreement; or
11. assault, interfere, deny service in any way or form to any other network, computer or node through the Service, or attempt to gain unauthorized access to any Services, or the accounts of other users, or computer systems or networks connected to the Services or

bypass any measures we may use to prevent or restrict access to the Services, or interfere with or disrupt servers or networks connected to any Services.

4.4 Open Source. The Open-Source code components that are included with the Software are redistributed by Aura under the terms of the applicable Open-Source Code license for such components. If there is an inconsistency between the Open-Source Code license, and these Terms, the Open-Source Code license controls. Your receipt of Open-Source code components from Aura under these Terms neither enlarges nor curtails your rights or obligations defined by the Open-Source Code license applicable to the Open-Source Code components. Copies of the Open-Source Code licenses for the Open-Source Code components that are included with the Software are included with or referenced in the Software's Documentation.

4.5 Availability. You may use the Software solely to access the Services. A computer or other equipment enabled to access the Internet (a "Device") is required to utilize the Services. You are solely responsible for ensuring that your Device is sufficient and compatible for use with the Service and complies with all System Requirements. The speed and quality of the Services may vary and the Services are subject to unavailability, including emergencies, third-party service failures, transmission, equipment or network problems or limitations, interference, signal strength, and maintenance and repair, and may be interrupted, refused, limited or curtailed.

4.6 Termination. Upon expiration or any termination of these Terms, you must stop using the Services and destroy all copies of the Software and any associated documentation in your possession.

Part 5. Additional Terms for Residents of Canada

This section sets out additional information for residents of Canada.

5.1 Subscription Terms, Payment, Renewals, and Cancellation. The term of your subscription (whether it is yearly or monthly) and your payment terms (both the amounts you pay, and when you pay them) will be set out before you purchase your subscription. For monthly subscriptions, your subscription continues until cancelled in accordance with Section 1.2 above.

5.1.1 Renewals for individuals residing outside of the province of Quebec:

- Yearly subscriptions: We will send you notice as required by law to inform you that your subscription will be automatically renewed, unless you cancel it. If you do not cancel, your subscription will be automatically renewed in accordance with Section 1.2 above. 5.1.2

5.1.2 Renewals for individuals residing in the province of Quebec:

- Yearly subscriptions:

Yearly Subscriptions automatically renew on a month-to-month basis at the end of your yearly Subscription. We will send you notice as required by law to inform you that your subscription automatically renews on a month-to-month basis at the end of the first year, unless you cancel it. If you do not cancel, your Subscription will be automatically renewed on a month-to-month basis at the price shown in your account dashboard or otherwise communicated to you by us (“Renewal Price”) unless (a) you or we have canceled your Subscription at least one (1) day prior to the commencement of your next Billing Cycle or in accordance with Section 1.4 above, or (b) an event under Section 1.6 occurs in which case we will give you prior notice according to that Section. We will email you in advance to let you know your Subscription is due for renewal and provide the renewal price in your account dashboard. You may cancel your Subscription by contacting us via the phone number in your account dashboard, which at the time of publishing is 1-833-552-2123, or by logging into your account. Once we or you have canceled your Subscription, your recurring subscription fees for the Services will no longer be charged to the payment method we have on file for your account, and your Subscription will remain active only until the end of the current Billing Cycle.

5.2 Amendments. We may amend, modify, or update all or any portions of the Terms or the Services at any time and from time to time, in our sole discretion, and including without limitation our prices, payment terms, warranties and cancellation policies, subscription terms, term structure and the nature, content, and features of our Services. If we do so, we’ll let you know either by posting the updated Terms on our website or through other communications. Where required by law or at our discretion, we will send a message to the email address (or other contact information at our discretion) associated with your account, setting out the new clause, or the amended clause and the clause as it read formerly, and the date of the coming into force of the amendment, at least thirty (30) days in advance of the change. Where required by law, or at our discretion, if you do not agree with any modification, then you may terminate the Services, without cost, penalty or cancellation indemnity. You may do so in accordance with the notice, and for individuals residing in Quebec, by sending us notice to that effect no later than thirty (30) days after the amendment comes into force.

Archived Versions

Current: October 9, 2024: Updates include revisions to arbitration agreement, governing law, service credits, and licensing agreements to customer data; Reorganized for great clarity

March 13, 2024: Updating terms for payment cycle changes

February 1, 2024: Updates to Terms of Service and Privacy Policy to reflect new regions of the Aura services

September 1, 2023: Updates to Term of Service and Privacy Policy to reflect new capabilities of the Aura services

March 4, 2023: Added new features such as Call Assistant

July 10, 2022: Added new features such as Parental Controls, clarified other terms

June 14, 2022: Updated Money Back Guarantee Terms

January 31, 2022: Minor updates to billing and other terms

January 1, 2022: Changed entity name to Aura Sub, LLC

August 16, 2021: Modified Free Trials (Section 2.4) and certain Service-Specific Terms.

June 8, 2021: Initial launch version of these Terms
