# Supplementary material for: Technology Effects and Child Health: Wellness Impact and Social Effects (TECHWISE): Protocol for a Prospective, Observational, Real-World Study
Source: JMIR Res Protoc. 2025 Jun 19;14:e69358. doi: 10.2196/69358 (PMC12226774; doi:10.2196/69358)
Supplement: Multimedia Appendix 4 [file resprot_v14i1e69358_app4.pdf]

Checklist for Reporting Results for Internet E-Surveys (CHERRIES)

Given the electronic administration of our outcome measures in the TECHWISE study, we have elected to follow the CHERRIES reporting guidelines. The checklist is below with information on where to access the relevant information, as well as any deviations from the checklist.

Item Category	***Checklist Category***	***Description/Location in Manuscript***
Design	*Describe Survey Design*	*Abstract, Methods*
IRB (Institutional Review Board) approval and informed consent process	IRB Approval	*Methods: Ethics Considerations and Approval*
	Informed Consent	*Methods: Ethics Considerations and Approval*; copies of informed consent and assent included in Appendix
	Data Protection	*Methods: Digital Activity Data Collection*; *Privacy Policy and Terms and Conditions included as Supplemental Material*
Development and pre-testing	Development and testing	All surveys used in the study were obtained with appropriate licensing/permissions; As per the SOPs for the study team and electronic data capture vendor, All surveys used in the study were built in an electronic format and tested extensively for usability and accuracy prior to sending to participants
Recruitment process and description of the sample having access to the questionnaire	Open survey versus closed survey	*Methods: Study Enrollment;* All surveys used in the study are only available to verified and enrolled participants and their caregivers; Individuals whose participation has not been verified by the study

		team are unable to access surveys
	Contact mode	*Methods: Study Recruitment, Study Enrollment*
	Advertising the survey	*Methods: Study Recruitment*
Survey administration	Web/E-mail	*Methods: Study Procedures*; surveys are shared with participants via a unique access link sent to either email or text
	Context	*Methods: Study Procedures;* Only enrolled and eligible participants are able to receive links for study surveys
	Mandatory/voluntary	*Methods: Study Procedures*; All aspects of the study are voluntary. However, compensation is available based on completing all surveys available at a given time point (monthly or daily surveys)
	Incentives	*Methods: Study Design; Methods: Table 1*
	Time/Date	Abstract; Results
	Randomization of items or questionnaires	*Methods: Study Outcomes*; Order of administration of surveys is fixed/not-rendomized
	Adaptive questioning	*Methods: Study Outcomes*; One of the study outcomes (K-CAT) is an adaptive measure. All other outcomes are fixed.
	Number of Items	*Methods: Study Outcomes*
	Number of screens (pages)	The number of items/screen is dependent on the device used by the participant to complete surveys. Surveys are designed to be administered via a

		browser that will have different resolution on a computer monitor/laptop, versus a mobile device. Survey administration is mobile-optimized for a clean interface with users completing surveys on phones or tablets.
	Completeness check	Surveys are automatically scored and participants are required to complete all items before moving forward, but are allowed to skip/not anser questions.
	Review step	Participants are able to move back and forth within a specific survey. Once a survey is submitted, however, it is not possible for the participant to change.
Response Rates	Unique site visitor	Due to the nature of the consent and enrollment process, all participants and caregivers for this study will be verified and data from them will be uniquely accessed
	View rate (Ratio of unique survey visitors/unique site visitors)	Although we will collect data on how many unique visitors visit the study landing page and express interest, only consented and verified participants will be enrolled and complete measures
	Participation rate (Ratio of unique visitors who agreed to participate/unique first survey page visitors)	Detailed information/consort diagrams showing consented participants and flow through the study will be presented in any subsequent analyses/presentations/paper s
	Completion rate (Ratio of users who finished the survey/users who agreed to participate)	Detailed information/consort diagrams showing consented participants and flow through the study will be presented in any subsequent analyses/presentations/paper

		s
Preventing multiple entries from the same individual	Cookies used	Cookies are not used in this study since all participants will be consented and eligibility verified. A participant may not enroll more than once. Parents/caregivers with multiple children will be allowed to enroll with separate children.
	IP check	We will not check IP addresses, but will verify parent and participant status with government issued identification.
	Log file analysis	The structure of our verification procedures and the nature of the electronic data capture system will ensure that multiple entries from the same participant are entered
	Registration	*Methods: Study Procedures*; Participants who consent and whose eligibility is verified will be sent unique links for survey completion
Analysis	Handling of incomplete questionnaires	Participants will be required to complete each survey before moving on to the next one; Methods for handling missing questionnaires from some participants will be described in all subsequent analyses/presentations/paper s
	Questionnaires submitted with an atypical timestamp	We will gather information about time and duration of completions and will review for unusual patterns
	Statistical correction	Statistical correction methods will be applied depending on the specific nature of the questions/analyses to be

		conducted.
